# Prophage-Derived Molecules as Anti-*Vibrio* Agents in Aquaculture: Mechanisms of Biofilm Control and Virulence Suppression

**DOI:** 10.3390/antibiotics15090874

**Published:** 2026-09-07

**Authors:** Nazia Tabassum, Aqib Javaid, Abirami Karthikeyan, Minji Kim, Young-Mog Kim, Fazlurrahman Khan

**Affiliations:** 1 Research Center for Marine Integrated Bionics Technology, Pukyong National University, Busan 48513, Republic of Korea; nazia99@pukyong.ac.kr (N.T.); minjikim@pknu.ac.kr (M.K.); 2 Marine Integrated Biomedical Technology Center, The National Key Research Institutes in Universities, Pukyong National University, Busan 48513, Republic of Korea; 3 Interdisciplinary Program of Marine and Fisheries Sciences and Convergent Technology, Pukyong National University, Busan 48513, Republic of Korea; aqibj@pukyong.ac.kr; 4 Industry 4.0 Convergence Bionics Engineering, Pukyong National University, Busan 48513, Republic of Korea; abirami@pukyong.ac.kr; 5 Department of Food Science and Technology, Pukyong National University, Busan 48513, Republic of Korea; 6 Ocean and Fisheries Development International Cooperation Institute, Pukyong National University, Busan 48513, Republic of Korea; 7 International Graduate Program of Fisheries Science, Pukyong National University, Busan 48513, Republic of Korea

**Keywords:** *Vibrio*, prophage, endolysin, depolymerase, biofilm, quorum sensing, phage therapy, aquaculture, antimicrobial resistance, combination therapy

## Abstract

Vibriosis causes substantial losses in marine and brackish-water aquaculture, while increasing antimicrobial resistance limits the effectiveness of conventional antibiotic treatment. Biofilm formation and quorum-sensing-controlled virulence further contribute to the persistence of pathogenic *Vibrio* species. Prophages integrated into *Vibrio* genomes encode proteins and regulatory elements that may provide alternative approaches for controlling these pathogens. This review evaluates endolysins, polysaccharide depolymerases, nucleases, holins, spanins, tailocins, regulatory proteins, and small RNAs associated with prophages and related phages. The available evidence was classified according to molecular origin to distinguish validated prophage-derived molecules from those obtained from temperate or lytic phages and from molecules characterized in non-*Vibrio* bacteria. Endolysins and depolymerases can disrupt bacterial cells and biofilm matrices, whereas prophage regulatory elements may influence quorum sensing, adhesion, motility, toxin production, and secretion systems. However, the evidence directly supporting prophage-derived anti-*Vibrio* agents remains limited. Most experimentally demonstrated activity has been reported for endolysins and one validated depolymerase, and many of these molecules originated from lytic rather than temperate phages. No direct *Vibrio*-specific evidence is currently available for phage-derived nucleases, holins, spanins, or tailocins as isolated control agents. Prophage genomes nevertheless provide an extensive source of regulatory and antimicrobial candidates for further investigation. Progress toward aquaculture application will require experimental validation, effective delivery methods, safety assessment, scalable production, and clear regulatory standards.

## 1. Introduction

Aquaculture is one of the fastest-growing food-producing sectors and a central pillar of global food security, but its intensification has amplified the risk of infectious disease outbreaks, which currently cost the industry an estimated USD 6 billion annually [1]. Among bacterial pathogens, members of the genus *Vibrio* stand out as the dominant cause of losses in marine and brackish-water culture systems. Vibriosis affects a broad range of finfish, penaeid shrimp, molluscs, and crustacean larvae, and its impact on global shrimp supply is severe enough to influence long-term prices and drive geographic relocation of production [2].

The clearest illustration is acute hepatopancreatic necrosis disease (AHPND), caused by toxin-producing strains of *Vibrio parahaemolyticus* and related species, which emerged in China and Vietnam in 2010 before spreading across South-East Asia and the Americas [3]. Between 2010 and 2016, combined losses attributed primarily to AHPND in China, Malaysia, Mexico, Thailand, and Vietnam were estimated at over USD 44 billion [3]. This figure is not directly comparable with the estimate above, because it aggregates farm-gate losses together with downstream contractions in feed sales and exports across five countries and a single disease complex, rather than counting direct disease costs across global aquaculture. Over the same period, shrimp production in affected regions fell to roughly 60% of pre-outbreak levels [4]. *Vibrio harveyi*, the etiological agent of luminous vibriosis, adds a further burden in penaeid shrimp and marine finfish through eye lesions, muscle necrosis, tail rot, and mass mortalities in hatcheries [5]. At the farm level, the impact is directly measurable: a bioeconomic study of net-cage-cultured Asian seabass in Peninsular Malaysia attributed 16.2% of mortality to vibriosis and estimated that the disease alone accounts for about 7% of total production cost per kilogram [6].

Conventional mitigation of vibriosis has relied heavily on prophylactic and therapeutic use of antibiotics [7], but this approach has produced diminishing returns and mounting collateral costs. Antibiotic and disinfectant regimens applied against AHPND have delivered only limited success while altering host gut microbiota and immunity and selecting for resistance in the target pathogens [4]. AHPND-causing *V. parahaemolyticus* isolates from Mexico now carry the *tetB* tetracycline-resistance determinant, and *V. campbellii* strains from China have been reported with multiple resistance genes [4].

More broadly, antimicrobials introduced across aquaculture, agriculture, and clinical settings pollute the aquatic environment, turning it into a hotspot for the emergence and horizontal spread of resistance among vibrios [8]. Aquatic environments now function as major reservoirs of antimicrobial-resistance genes, and most *Vibrio* species examined display resistance to β-lactams, aminoglycosides, tetracyclines, sulphonamides, quinolones, and macrolides [9]. The rising incidence of multidrug-resistant *Vibrio* isolates poses a dual threat to aquaculture sustainability and public health, particularly in low- and middle-income countries where seafood is a critical protein source [10]. These pressures have made non-antibiotic control strategies a research priority rather than an academic curiosity.

One reason antibiotics underperform against *Vibrio* infections is that the pathogens do not persist as isolated planktonic cells. Vibrios are natural inhabitants of aquatic environments and readily form matrix-enclosed, surface-associated biofilms whose assembly depends on flagella, pili, exopolysaccharide biosynthesis, two-component regulators, quorum sensing, and cyclic-di-GMP signaling [11]. Biofilms are not a passive lifestyle only: they act as reservoirs from which pathogenic vibrios launch infections. In *Vibrio tapetis*, for example, biofilm-associated cells strongly induce Type VI secretion machinery and AI-2/LuxO quorum sensing, directly linking the sessile state to virulence expression [12].

Quorum sensing itself is a central regulatory node coupling cell density to virulence and biofilm dynamics across *Vibrio fischeri*, *V. harveyi*, *V. cholerae*, and *V. vulnificus* [13]. In *V. cholerae*, the master quorum-sensing regulator HapR represses biofilm formation both by lowering intracellular c-di-GMP and by directly repressing the biofilm activator *vpsT*, integrating extracellular signals with intracellular second messengers [14]. Because biofilm matrices restrict antibiotic penetration and quorum sensing coordinates the timing of toxin release, adhesion, and secretion-system deployment, disrupting these two networks (rather than simply killing planktonic cells) has emerged as a rational anti-*Vibrio* strategy that also imposes weaker selective pressure for resistance.

Bacteriophages have been investigated as alternatives to antibiotic treatment of vibriosis, for prevention as well as therapy in aquaculture [15]. Most of this work has used lytic phages, whereas temperate phages that integrate into *Vibrio* genomes as prophages have received less attention. Prophages are widespread in marine Vibrios and constitute a major reservoir of accessory functions: screening of environmental *Vibrio* isolates from the North Sea recovered inducible prophages from 40% of *V. parahaemolyticus* strains and from *V. cholerae*, with genomes carrying integrases, transposases, and mobile elements that shape host phenotype and evolution [16].

Prophage genomes encode proteins that have been examined for antibacterial use. Phage-derived lytic enzymes and proteins have been studied as tools to reduce dependence on antibiotics [7], and this work has recently been extended to *Vibrio*. Systematic mining of NCBI recovered 37 phage lysins targeting *V. parahaemolyticus*, three of which cleared over 90% of preformed biofilms within 48 h, reversed partial antibiotic resistance, and acted synergistically with gentamicin [17]. Bioinformatic analysis further shows that 79.4% of cultured and 46.2% of uncultured *Vibrio* phages encode putative polysaccharide depolymerases, a class of enzymes that degrade *Vibrio* surface polysaccharides, disrupt biofilms, and delay the emergence of resistance when combined with their parental phage [18]. These results indicate that prophage genomes contain antibiofilm and anti-virulence candidates that have not yet been examined systematically for vibriosis control in aquaculture.

Prophage-derived molecules (endolysins, polysaccharide depolymerases, DNases, holins and spanins, tailocins, and regulatory proteins and small RNAs) reported to act against pathogenic *Vibrio* species are examined here and distinguished from molecules obtained from virulent phages, with which they are frequently conflated in the secondary literature. The objectives are to: (1) summarize prophage biology and distribution in the major aquaculture-relevant *Vibrio* species, (2) classify the therapeutically relevant molecular families and their mechanisms, and assign every reported molecule an explicit provenance, (3) examine how these molecules disrupt biofilm assembly and suppress virulence programs, (4) evaluate translational applications in aquaculture, from hatchery water treatment to feed-based delivery, (5) survey combination strategies with antibiotics, probiotics, biosurfactants, and nanoparticle carriers, and (6) identify the key stability, resistance, biosafety, regulatory, and translational challenges that must be addressed before prophage-derived anti-*Vibrio* agents can move from laboratory studies into commercial aquaculture practice.

A major gap in the current literature is the frequent grouping of prophage-derived molecules with products obtained from virulent phages. This makes it difficult to determine which antibiofilm and anti-virulence activities are supported specifically by molecules encoded within the prophage compartment.

### 1.1. Why Prophage-Derived Molecules Are Treated as a Distinct Class

In this review, prophage-derived refers only to molecules encoded by genetic elements that are demonstrated to reside within bacterial genomes as prophages or satellite prophages. The broader term phage-derived includes products originating from both temperate and virulent phages.

Because prophages and virulent phages both encode lysis machinery, the case for treating prophage cargo as a separate category needs to be made rather than assumed. Three arguments support it. The first concerns the discovery route. Prophages are recovered computationally from sequenced bacterial genomes, without phage isolation, host culture, or plaque assay, and the resource is already large: 5674 prophage-like elements have been cataloged across 1874 *Vibrio* genomes spanning 64 species [19], 55 elements across 28 *V. anguillarum* strains [20], and 13 curated prophages across 55 *V. harveyi* genomes [21]. The second concerns cargo type. Virulent phages encode structural and lysis functions, whereas prophages also encode the regulatory apparatus of lysogeny, including antirepressors, autoinducer-responsive transcription factors, superinfection-exclusion proteins, and Hfq-binding small RNAs, none of which has an equivalent in the virulent-phage repertoire. This regulatory cargo is the clearest distinguishing feature of the prophage compartment (Section 4.6 and Section 6). The third is definitional: tailocins are domesticated defective prophages, so no virulent phage produces them (Section 4.5).

One point should be conceded at the outset, because failing to state it has produced repeated misclassification in this field. For enzymes, the distinction is largely immaterial. An endolysin encoded by a temperate phage acts at the end of an induced lytic cycle and is mechanistically indistinguishable from an endolysin of a virulent phage, so there is no biochemical reason to expect prophage endolysins to outperform their virulent-phage counterparts. This is why the endolysin and depolymerase literature summarized in Section 4.1 and Section 4.2 is dominated by virulent isolates, and we label those entries accordingly rather than presenting them as prophage products. What genuinely distinguishes purified prophage-derived proteins from whole-phage therapy is a different set of properties: they do not replicate, cannot transduce host DNA, do not depend on receptor availability for potency, have a defined composition suitable for regulatory dossiers, and release no self-replicating agent into rearing water. Those properties, rather than the gene’s lysogenic origin, are the practical case for the class.

### 1.2. Scope, Literature Search, and Provenance Criteria

This is a narrative review rather than a systematic one, and its reporting was checked against the six items of SANRA, the Scale for the Assessment of Narrative Review Articles [22]. Literature was identified through iterative searches of Web of Science, Scopus, PubMed, and Google Scholar, with forward and backward citation tracking from the key primary studies, combining prophage and phage terms with *Vibrio* and aquaculture terms, and with terms for the molecular classes and activities covered here. No formal screening protocol was applied, and no counts of records are reported because this is a qualitative synthesis rather than a systematic review or meta-analysis. Coverage is therefore representative rather than exhaustive, and statements that a molecule or class has not been reported for *Vibrio* reflect what this search identified. Entries qualified for Tables 1–3 only if the source reported an experimentally measured outcome, and each was assigned one of five provenance categories used throughout: experimentally validated prophage-derived, where the molecule is encoded by an element demonstrated to reside in a bacterial genome as a prophage or satellite prophage; temperate-phage-derived, where the parent phage is documented as temperate but was characterized as a free phage; lytic-phage-derived, where the primary source describes the parent phage as virulent; hypothetical, where the molecule or activity is predicted rather than isolated and assayed; and comparative example, for work performed outside the genus *Vibrio*. Negative and null results are reported alongside positive findings.

## 2. Prophages in Pathogenic *Vibrio* Species

### 2.1. Prophage Biology: Lysogeny, Induction, and the Lytic-Lysogenic Switch

Temperate bacteriophages can follow one of two developmental pathways after infecting a susceptible *Vibrio* host. In the lytic pathway, phage genomes replicate rapidly and progeny particles are released by cell lysis; in the lysogenic pathway, viral replication and lytic functions are suppressed and the phage persists as a prophage (either integrated into the bacterial chromosome by host- or phage-encoded recombinases, or maintained as an extrachromosomal plasmid- or telomere-like replicon) while the bacterium carrying it is termed a lysogen [23,24]. Once established, prophages can revert to lytic growth in a process called induction, typically triggered by perturbations that damage host DNA or otherwise activate stress responses [23].

The molecular circuitry underlying this decision is essentially a genetic switch that senses infection or damage inputs and commits the cell to protection or death, closely paralleling the logic of the classical Lambda paradigm [25]. In vibrios, the input side of the switch is particularly rich. Sub-inhibitory concentrations of a wide range of antibiotics (including fluoroquinolones, aminoglycosides, tetracyclines, and chloramphenicol) induce the SOS response in *Vibrio cholerae*, in contrast to *Escherichia coli*, where several of these classes are silent [26]. Comparative genomics of the *Vibrionaceae* has shown that the SOS regulon is strongly linked to pathogenicity and to the mobilization and dissemination of antibiotic-resistance determinants, and that core SOS genes are conserved on the large chromosome while specialized additions accumulate on smaller chromosomes and plasmids [27]. Prophage induction can also be regulated by host cell-density signals: quorum sensing represses induction of the ϕH20-like prophage of *V. anguillarum* at high cell density, so that phage particle production is up to eightfold higher in quorum-sensing-deficient mutants [28]. Environmental factors and host quorum-sensing signals therefore both feed into the lysis-lysogeny decision, and the ability of prophages to remain silent or reactivate rapidly makes them durable reservoirs of bioactive genes in aquaculture settings [24].

### 2.2. Occurrence and Diversity of Prophages in Aquaculture-Relevant Vibrio Species

Prophage-like elements are ubiquitous across the genus. A database of 5674 prophage-like elements assembled from 1874 *Vibrio* genome sequences spanning 64 species revealed that elements encoding putative virulence and antibiotic-resistance traits are widely distributed among both clinical and environmental isolates, and that 45% of *Vibrio* species harbor a complete Inoviridae prophage encoding the zonula occludens toxin (Zot) originally described in *V. cholerae* [19]. Table 1 compiles representative prophages characterized in pathogenic *Vibrio* species, together with their genome features and encoded cargo. Phage names in this review follow the usage of the primary sources. Because the morphology-based families Myoviridae, Siphoviridae, and Podoviridae and the order Caudovirales were abolished by the International Committee on Taxonomy of Viruses in 2022 [29], these names are retained here only where they appear in the cited work, and the corresponding morphotype terms (myovirus, siphovirus, podovirus) are used in new descriptive text; currently valid taxa such as Inoviridae, Peduoviridae, and Ackermannviridae are used as ratified.

(1) *Vibrio cholerae* provides the paradigmatic example. The structural genes for cholera toxin are carried by CTXΦ, a filamentous phage related to coliphage M13 whose genome either integrates chromosomally or replicates as a plasmid, and which uses the toxin-coregulated pilus (TCP) as its cell-surface receptor [30]. Its discovery established that the emergence of toxigenic *V. cholerae* depends on the horizontal transfer of a phage-encoded cassette, and CTXΦ remains the most thoroughly studied virulence-linked *Vibrio* prophage [30,31].

(2) *Vibrio parahaemolyticus* carries diverse prophages. Screening of North Sea isolates recovered inducible prophages from 40% of *V. parahaemolyticus* strains [16], and single-strain genomic analysis of one of these isolates characterized the HP1-like Peduoviridae prophage VPS05ph1, excisable by mitomycin C, alongside a second co-resident element [32]. Filamentous phages of *V. parahaemolyticus* have also been characterized, including the K38-antigen-restricted phage Vf33 and the inducible inovirus vB_VpaI_VP-3218, which carries putative zonula occludens toxin genes, paralleling the CTXΦ paradigm [33,34].

(3) *Vibrio harveyi* genomes are heavily colonized by prophages. Comparative analysis of 55 globally sourced *V. harveyi* genomes identified 13 well-curated tailed prophages spanning eight subfamilies and nine genera, with distinct parasitic strategies including Mu-type transposition, site-specific recombination, and plasmid-like non-integrated states [21]. The archetype of virulence-converting *V. harveyi* prophages is VHML (*Vibrio harveyi* myovirus-like), a 43 kb myovirus whose presence induces virulence in laboratory-passaged hosts and whose genome contains a putative ADP-ribosylating toxin gene consistent with phage-mediated toxin conversion [35].

(4) *Vibrio anguillarum* hosts one of the most functionally validated prophage repertoires described to date. Sequencing of 28 *V. anguillarum* strains revealed 55 different prophage-related elements, with a shared p41 sequence dynamically exchanged among 17 strains; chemical and spontaneous induction yielded 42 phage isolates, and induced particles could re-lysogenise naïve *V. anguillarum* strains, demonstrating an active phage-bacterium exchange within the species [20].

(5) *Vibrio alginolyticus* has been shown to carry filamentous phages that resemble CTXΦ. In the Kiel *V. alginolyticus* ecotype, eight strains isolated from different pipefish share an identical prophage repertoire, including two actively replicating Inoviridae, one of which encodes Zot- and Ace-like proteins homologous to *V. cholerae* CTXΦ toxins; coverage of this phage correlated positively with virulence in controlled infections [36].

(6) *Vibrio vulnificus* was long considered an outlier because its virulence had not been mapped to a prophage. Isolation of the temperate myovirus PV94 from a biotype 1 clinical isolate showed a modular 33.8 kb genome whose immunity/integrase/replication modules resemble *V. cholerae* κ phages while structural modules match a prophage from *V. furnissii*, underlining the extent of horizontal exchange within the genus [37].

(7) *Vibrio campbellii*. Related work on the shrimp pathogen *V. campbellii* ATCC BAA-1116 (formerly *V. harveyi* BAA-1116) identified two mitomycin- and heat-inducible Myoviridae prophages resembling ΦHAP-1 and phage κ, further extending the prophage catalog to the aquaculture-relevant clade [38].

**Table 1:** Prophages and prophage-like elements characterized in *Vibrio* species, including host, element name, genome features, and encoded cargo. Two entries are retained for comparison and are not prophages of a pathogenic *Vibrio*: the virulent phage KVP40, included because it is the direct comparator in the biofilm study cited, and the element of the non-pathogenic marine diazotroph *V. diazotrophicus*, included for its biofilm biology. The final entry concerns free environmental transducing phages rather than resident prophages. Family names follow the primary sources; morphology-based families were abolished by the ICTV in 2022 [29].

Host	Prophage Name	Genome Family Features	Encoded Cargo	Reference
*V. cholerae*	CTXΦ (cholera toxin phage)	Filamentous phage (Inoviridae) related to coliphage M13; ~7 kb ssDNA; integrates chromosomally or replicates as a plasmid; uses toxin-coregulated pilus (TCP) as cell-surface receptor	Cholera-toxin structural genes (*ctxAB*); their horizontal transfer defines toxigenic *V. cholerae* emergence, the paradigmatic virulence-linked *Vibrio* prophage	[30]
*V. cholerae*	K139 (κ/kappa)	Temperate myovirus (κ family); integrates into the *V. cholerae* genome	Predatory phage particles that kill non-lysogens; in chitin estuary microcosms, K139 lysogens kill non-lysogens through induced phage particles and simultaneously acquire environmental DNA by natural transformation, enhancing HGT and lysogen fitness	[39]
*V. parahaemolyticus*	VPS05ph1 HP1-like Peduoviridae) and a co-resident second element	HP1-like Peduoviridae prophages characterized at the genome level; mitomycin-C-inducible; polylysogeny reported in the source strain	Discovery in a North Sea isolate; 40% of screened *V. parahaemolyticus* strains carried inducible prophages, demonstrating widespread lysogeny in the species	[16,32]
*V. parahaemolyticus*	VP882 (temperate vibriophage)	Temperate phage; encodes VqmA quorum-sensing autoinducer receptor and Qtip antirepressor as a QS-driven lysis/lysogeny switch	Phage-encoded VqmA homolog binds host QS autoinducer DPO and, together with antirepressor Qtip, sequesters the phage repressor to launch the lytic program; VqmA also activates the host VqmR regulon, reprogrammable to produce phages that lyse on user-defined QS cues	[40]
*V. parahaemolyticus* CHN25	Prophage encoding *VpaChn25_0734*	Chromosomal prophage cluster, identified through targeted deletion studies	*VpaChn25_0734* enhances swimming motility, cell-surface hydrophobicity, biofilm formation, and cytotoxicity toward human intestinal epithelial cells; deletion produces growth defects at low temperature and pH, and knockdown of the wider prophage cluster reduces ecological persistence; prophage acts as a fitness modulator rather than as a toxin carrier	[41]
*V. harveyi* (55 globally sourced strains)	13 well-curated tailed prophages	Span 8 subfamilies and 9 genera of tailed phages; distinct parasitic strategies including Mu-type transposition, site-specific recombination, and plasmid-like non-integrated states	Comparative genomics of 55 *V. harveyi* genomes demonstrates that *V. harveyi* genomes are heavily colonized by phylogenetically diverse prophages using multiple lifestyles	[21]
*V. harveyi*	VHML (*Vibrio harveyi* myovirus-like)	43 kb myovirus; temperate; contains a putative ADP-ribosylating toxin gene	Induces virulence in laboratory-passaged *V. harveyi* hosts; phage-mediated toxin conversion analogous to CTXΦ, the archetype of virulence-converting *V. harveyi* prophages	[35]
*V. campbellii* ATCC BAA-1116	Two intact Myoviridae prophages (one ΦHAP-1-like on chromosome I; one *Vibrio*-phage-κ-like on chromosome II)	Both intact prophages; inducible by mitomycin C and by heat stress (50 °C, 30 min); heterogeneous induction across the population revealed by two-gene promoter reporters	Both prophages produced morphologically distinct infectious phage particles upon induction, documenting dual-lysogeny and dual-inducibility in a hatchery-relevant *Vibrio* species	[38]
*V. anguillarum* (28 sequenced strains)	55 prophage-related elements clustered into 7 groups; shared prophage p41 in 17 strains	42 phage isolates from chemical + spontaneous induction; multiplex-PCR classification; host range strongly depends on the strain used for proliferation (host-regulated phenotype)	Prophage ϕVa_90-11-287_p41 lysogenizes *V. anguillarum* Ba35 and is then spontaneously released, providing direct evidence of efficient prophage transfer among *V. anguillarum* populations and documenting broad prophage-driven genetic diversity in the species	[20]
*V. anguillarum*	ϕH20-like temperate prophage (Siphoviridae)	Repeatedly identified as free phages and as prophages across geographically distinct areas, induction is gated by the host quorum-sensing state	QS-locked Δ*vanT* (low-cell-density) host produced ~8-fold more phage particles per cell than high-cell-density Δ*vanO*; prophage stimulates biofilm formation at low cell density, while high-density state reduces induction and represses biofilm, direct QS-prophage-biofilm interplay	[28]
*V. alginolyticus* (Kiel pipefish ecotype)	Inoviridae prophages (CTXΦ-like filamentous phage lineage)	Eight strains isolated from different pipefish share an identical prophage repertoire, including two actively replicating Inoviridae	One filamentous prophage encodes Zot- and Ace-like proteins homologous to *V. cholerae* CTXΦ toxins; coverage of this phage correlated positively with virulence in controlled pipefish infections	[36]
*V. anguillarum* (and environmental deep-sea vibrios)	Inoviridae prophages encoding Zot/Ace homologs	Filamentous CTXΦ-like phages detected across *V. anguillarum*, deep-sea isolates, and *V. alginolyticus*	Zot and Ace toxin homologs suggest a genus-wide role for filamentous prophages in virulence acquisition; a CRISPR spacer in *V. anguillarum* PF7 matches the *zot* gene of an inovirus resident in strain PF4, evidence of ongoing host-filamentous-phage arms-race dynamics	[19]
*V. vulnificus* (biotype 1 human isolate)	PV94	Temperate myovirus; linear dsDNA 33,828 bp with 5′-protruding ends; mitomycin-C-inducible; modular mosaic genome (immunity/integrase/terminase similar to *V. cholerae* κ phages; structural module similar to a prophage in *V. furnissii* NCTC 11218)	First genome-sequenced temperate phage from a human *V. vulnificus* isolate; mosaic architecture indicates high horizontal genetic exchange within the *Vibrio* genus, providing a route for *V. vulnificus* to acquire virulence-associated genes from other species	[37]
*V. diazotrophicus* (marine non-cyanobacterial diazotroph; not a pathogen, included for comparison)	Myoviridae prophage (unnamed)	Myovirus prophage; expression is induced under anoxic and biofilm-forming conditions; spontaneously excises from the host genome and forms intact infective phage particles	Phage-mediated host cell lysis increases biofilm production compared with a prophage-free derivative and releases dissolved organic carbon and ammonium. Prophage links marine phage-diazotroph interplay to biofilm architecture and nutrient cycling	[42]
*V. campbellii* HY01 (emerging shrimp pathogen)	HY01	Long-tailed phage (Siphoviridae); dsDNA 41,772 bp; G + C 47.45%; 60 ORFs (31 functionally predicted); mitomycin-C-inducible	Distantly related to *V. anguillarum* phage Va_PF430-3_p42 but differs in genome size and gene organization; two CRISPR-spacer matches in *Vibrio* spp. suggest that HY01 evolution is driven by horizontal genetic exchange between Gammaproteobacterial families, the first genome-scale prophage from an emerging luminous-vibriosis shrimp pathogen	[43]
*V. parahaemolyticus* (K38-antigen strains)	Vf33	Filamentous phage (~1400 nm long, 7 nm wide); ssDNA circular 8.4 kb genome; buoyant density 1.292 g/cm^3^ in CsCl; lytic activity resistant to heating below 80 °C, diethyl ether, acetone and methanol but sensitive to chloroform; genome converted to dsDNA replicative form in host	Historic characterization (1984) of a strictly K38-antigen-restricted filamentous phage of *V. parahaemolyticus* establishes filamentous-phage tropism to specific surface antigens as a driver of strain-level susceptibility	[33]
*V. parahaemolyticus* VN-3218 (North Sea isolate)	vB_VpaI_VP-3218	Filamentous phage of the Inoviridae (Saetivirus-related); complete genome 11,082 bp; integrates into the chromosomal host genome and replicates as an episome; mitomycin-C-inducible	Carries host-related regions absent from similar phage genomes and potential zonula occludens toxin (Zot-like) genes, implicated in the emergence of new pathogenic strains in the warming North Sea; adds a second CTXΦ-lineage filamentous prophage to the aquaculture-relevant catalog	[34]
*V. alginolyticus* AP-1 (emerging marine pathogen)	Vaf1 (filamentous prophage)	Non-lytic filamentous phage of the Inoviridae family; replicates without killing its host; establishes long-term persistence; genome resident in the AP-1 chromosome	Presence of Vaf1 significantly increases biofilm formation, swarming motility, and contact-dependent competition of *V. alginolyticus* AP-1; several phage genes were upregulated under low-nutrient conditions; in vivo zebrafish infection with ΔVaf1 was less lethal than wild-type, providing direct evidence that a filamentous prophage contributes to *V. alginolyticus* virulence	[44]
*V. cholerae* (non-O1/O139); free environmental phages, not resident prophages	Environmental CTX-transducing vibriophages	Naturally occurring vibriophages mobilize cholera-toxin (virulence) genes into non-O1/O139 strains	Vibriophages recovered from coastal waters transferred cholera-toxin genes from toxigenic O1 to non-O1/O139 *V. cholerae*, showing that environmental phages disseminate virulence determinants into non-O1 lineages and extending the CTXΦ virulence-transfer paradigm beyond the O1/O139 serogroups	[45]
*V. anguillarum* (comparative strain-level survey)	ϕH20 (siphovirus prophage) and KVP40 (virulent myovirus, applied externally; not a prophage)	Contrasting phage lifestyles produce strain-specific outcomes; the resident ϕH20 prophage reduced biofilm formation and free-living cells in strain BA35, whereas the virulent phage KVP40 stimulated aggregation and biofilm formation in strain PF430-3, creating spatial refuges from phage predation	Divergent effects on biofilm architecture and phage-resistance dynamics between two closely related *V. anguillarum* strains highlight that phage-host interplay in vibriosis control cannot be generalized across strains and requires per-strain characterization	[46]

## 3. Beneficial and Detrimental Roles of Prophages

### 3.1. Virulence Evolution

The clearest role of *Vibrio* prophages is as vehicles for horizontal transfer of virulence determinants. Beyond CTXΦ, phages contribute additional accessory functions and participate in bactericidal selection processes that favor the emergence of new epidemic clones of *V. cholerae* [47]. The VHML prophage of *V. harveyi* directly converts recipient hosts to a more virulent phenotype through delivery of an ADP-ribosylating toxin gene [35,48], and Inoviridae prophages encoding Zot and Ace homologues have been detected in a wide range of environmental vibrios including *V. anguillarum*, deep-sea isolates, and *V. alginolyticus*, suggesting a genus-wide role for filamentous prophages in virulence acquisition [19,36]. A CRISPR spacer in *V. anguillarum* strain PF7 matching the *zot* gene of an inovirus resident in strain PF4 points to arms-race dynamics between hosts and their filamentous phages [19].

### 3.2. Stress Adaptation and Host Fitness

Not all prophage cargo is overtly toxic. The prophage-encoded gene *VpaChn25_0734* in *V. parahaemolyticus* CHN25 enhances swimming motility, cell-surface hydrophobicity, biofilm formation, and cytotoxicity toward human intestinal epithelial cells; its deletion produced growth defects at low temperature and pH, and knockdown of the wider prophage gene cluster reduced ecological persistence, showing that prophage genes can act as fitness modulators rather than only as toxin carriers [41]. Prophage-encoded superinfection exclusion systems may provide an additional benefit to the host. In *Pseudomonas*, a filamentous prophage-encoded protein modulates pilus dynamics through PilZ and prevents secondary phage infection without an apparent fitness cost [49]. Comparable activity has not yet been experimentally demonstrated in *Vibrio*, and this example is included only as mechanistic precedent.

### 3.3. Biofilm Modulation

Prophages actively remodel *Vibrio* biofilm behavior, and the direction of the effect is strain-dependent rather than uniform. In *V. anguillarum*, induction of the ϕH20-like prophage is repressed by quorum sensing at high cell density; at low density, spontaneous induction promotes biofilm formation, and comparison with prophage-free strains indicates that the H20-like element itself stimulates surface colonization, embedding the lysis-lysogeny switch within the host biofilm regulatory network [28]. In *V. diazotrophicus*, a myovirus prophage is induced specifically under anoxic and biofilm-forming conditions, and phage-mediated cell lysis raises biofilm biomass and releases dissolved organic carbon and ammonium, showing that prophage-driven lysis can seed biofilm matrix production rather than dismantle it [42]. The same element can nonetheless act in opposite directions in different genetic backgrounds: in a comparison of two *V. anguillarum* strains, ϕH20 reduced both biofilm formation and free-living cell numbers in strain BA35, whereas the virulent phage KVP40 stimulated aggregation in strain PF430-3 as a phage-avoidance response [46]. The apparent conflict between this result and the biofilm-stimulating effect reported in [28] is a genuine feature of the system rather than a discrepancy in the literature, since the two studies differ in host strain, in phage dose, and in whether the element was resident or applied externally. It follows that no general rule relates prophage carriage to biofilm phenotype, and that per-strain characterization is a prerequisite for any biofilm-control application, a constraint returned to in Section 9.2. More broadly, temperate phages can stabilize and promote biofilm growth through a combination of antagonistic killing and cooperative matrix release, a dynamic that has now been reported across marine bacteria [50].

These findings show that prophage induction cannot be regarded as uniformly beneficial for biofilm control. Its effect depends on the bacterial strain, prophage, environmental conditions, and stage of biofilm development. Candidate treatments should therefore be tested for their effects on both planktonic cells and biofilms in the target *Vibrio* strain. Purified recombinant molecules provide a more controlled approach because their application does not require deliberate induction of resident prophages.

### 3.4. Horizontal Gene Transfer

Finally, prophages are central drivers of *Vibrio* genome plasticity. HGT is a dominant force in the evolution of vibrios, and phages, together with plasmids, genomic islands, and integrative conjugative elements, account for much of the mobile-DNA repertoire that expands ecological and pathogenic niches in the genus [51,52]. Beyond classical transduction, prophage-encoded viral particles can act as weapons of neighbor predation: in chitin estuary microcosms, *V. cholerae* K139 lysogens kill non-lysogens through induced particles and simultaneously acquire environmental DNA by natural transformation, enhancing HGT and providing a fitness advantage [39]. This dual role (as reservoirs of virulence genes and as mobilizers of the surrounding genome) is what makes prophages both a threat to aquaculture (through the spread of toxin and resistance determinants) and, when their bioactive proteins are mined, a promising source of anti-*Vibrio* therapeutics, which the remainder of this review develops.

The same genetic compartment that supplies candidate antibiofilm and anti-virulence proteins is also the principal vehicle by which toxin genes, resistance determinants, and biofilm-promoting functions move between vibrios. This dual-use character has two practical consequences. First, it favors purified recombinant single proteins over whole temperate phages as the deployable product format, because a protein preparation carries no integrase, no packaging machinery, and no capacity for lysogenic conversion. Second, it makes genomic screening of the source element a design requirement rather than a regulatory formality. 

## 4. Classes of Prophage-Derived Molecules Reported as Antibacterial Agents

Every molecule described below, and every entry in Table 2 and Table 3, carries one of the five provenance categories defined in Section 1.2: experimentally validated prophage-derived, temperate-phage-derived, lytic-phage-derived, hypothetical, or comparative example. Applying this scheme substantially changes the shape of the evidence base. Of the molecules reported to act on *Vibrio*, only the vibriophage VP882 regulatory modules and the RS1 satellite-phage antirepressor RstC are experimentally validated prophage-derived agents; the endolysins and the single validated depolymerase are almost all lytic-phage-derived; and three of the six classes described below, namely phage nucleases, holins and spanins, and tailocins, have no *Vibrio*-specific molecule at all and are presented as gaps rather than as options.

### 4.1. Endolysins

Endolysins are peptidoglycan (PG) hydrolases expressed at the end of the phage lytic cycle to release progeny particles by degrading the bacterial cell wall from within, but when applied externally, they cause “lysis from without” and function as targeted antibacterials [53]. Most endolysins adopt a modular architecture, combining one or more catalytic domains (glycosidase, N-acetylmuramoyl-L-alanine amidase, endopeptidase, or lytic transglycosylase) with one or more cell-wall-binding domains that determine host specificity [53,54]. Because these enzymes act on a conserved, essential structure that has coevolved with the phage, resistance is thought to arise slowly, and engineered variants have opened applications spanning food safety, decontamination, and clinical antimicrobials [53].

Applying endolysins to Gram-negative *Vibrio* pathogens is complicated by the outer membrane (OM), which shields the underlying PG from externally added protein [55]. Two general strategies have emerged: pair natural endolysins with OM permeabilizers (EDTA, essential oils, cationic peptides), or engineer chimeric artilysins that fuse an OM-destabilizing module to the catalytic core [55]. Neither strategy transfers automatically to marine conditions. When the lytic-phage-derived, non-*Vibrio* endolysins KZ144 (from *Pseudomonas aeruginosa* phage φKZ) and LysPA26 were tested against *V. parahaemolyticus* in natural seawater, both retained muralytic activity, but neither synergized with EDTA or with oregano essential oil, and the pathogen subsequently metabolized the applied protein as a growth substrate [56]. This is a negative result rather than a dosing problem, and it is treated as such here and in Section 8.2: permeabilizer pairing is a matrix-specific andspecies-specific engineering question, not a general solution.

The best-characterized *Vibrio*-active endolysin is Lysqdvp001, encoded by phage qdvp001, which hydrolyses *V. parahaemolyticus* PG and shows a broader lytic spectrum than its parental phage [57]. The provenance of this lineage is frequently misdescribed: qdvp001 is a lytic broad-spectrum myovirus of 134,742 bp whose genome contains no identifiable holin gene and whose endolysin is homologous to that of the virulent *V. cholerae* phage ICP1 [7,57]. Lysqdvp001 and every derivative built on it are therefore lytic-phage-derived rather than prophage-derived. Its truncated catalytic domain, Plychap001, is stable across broad temperature, pH, and NaCl ranges, acts synergistically with EDTA, and efficiently eliminates *V. parahaemolyticus* biofilms on polystyrene surfaces [58]. Cationic-peptide chimeras built on the same scaffold (Lysqdvp001-15aa) achieve MIC/MBC of 0.2/0.4 mg mL^−1^ against *V. parahaemolyticus*, remove approximately 50% of preformed biofilms and inhibit more than 90% of biofilm formation [59], and the virtual-screening-designed artilysin sTHA-Lys removes more than 82% of mature biofilms while completely inhibiting new biofilm assembly [60]. Unmodified Lysqdvp001 has little intrinsic effect on preformed biofilms, and the reported antibiofilm activity of the parent enzyme is obtained in combination: paired with ε-poly-lysine it produced 3.75, 4.16, and 2.50 log_10_ CFU g^−1^ reductions in cod, shrimp, and oyster tissue respectively, a range of 2.50 to 4.16 log_10_ CFU g^−1^, together with 44 to 68% biofilm removal from polystyrene, glass, and stainless steel [61]. The oyster matrix gives the weakest result of the three, and quoting only the two stronger values overstates performance in the matrix most relevant to shellfish production.

Beyond the qdvp001 lineage, systematic mining is broadening the catalog, although it has not yet changed the provenance picture. Genome mining of 163 *Vibrio* phages recovered 168 putative endolysin genes and yielded LysMD30, the first *Vibrio*-phage-derived L-alanyl-D-glutamate peptidase, expressed in *Pichia pastoris*; the parent phage is not named, and its lifestyle is not reported, so its provenance is undetermined rather than prophage-derived. LysMD30 lacks intrinsic bactericidal activity but synergizes with colistin (FICI < 0.5) against *V. parahaemolyticus*, *V. harveyi*, and *V. alginolyticus*, reduces the colistin MIC fourfold, achieves more than 3 log reduction in biofilm viable counts on polystyrene and stainless steel within one hour, and delays colistin-resistance emergence [62]. The dependence on colistin is a substantive constraint rather than a technical detail. Colistin is listed by the World Health Organization among the highest-priority critically important antimicrobials for human medicine [63], plasmid-mediated *mcr* colistin resistance was first described from food-animal sources and is attributed substantially to agricultural use [64], and colistin was withdrawn as a feed additive in China in 2017 and is restricted in the European Union. A prophage-derived agent whose activity requires a critically important antimicrobial does not relieve the selection pressure that motivates the search for antibiotic alternatives, and the non-antibiotic permeabilizer routes discussed in Section 8.2 are better suited to aquaculture. Screening of 37 NCBI-derived phage lysins recovered three enzymes, again of unstated parent-phage lifestyle, that cleared over 90% of preformed *V. parahaemolyticus* biofilms within 48 h and acted synergistically with gentamicin [17]. Species-specific candidates include LysVPB, an unusually divergent, calcium-boosted 17 kDa endolysin against *V. parahaemolyticus* whose modular architecture allows domain swapping for chimeric design [65], LysVpKK5, an amidase_2 endolysin that retains approximately 50% activity in seawater and prefers alkaline pH [66], and LysVPMS1, which shows broad lytic activity against *V. parahaemolyticus* strains associated with AHPND [67]. As comparative examples from outside the genus, the endolysins LysAm24 (*Acinetobacter* phage AM24), LysAp22 (*Acinetobacter* phage Ap22), and LysECD7 (*E. coli* phage ECD7) remove Gram-negative biofilm biomass by attacking both peptidoglycan and matrix polymers, with LysAm24 binding extracellular DNA, LysAp22 disrupting acidic exopolysaccharides, and LysECD7 combining both activities [68]. None of these three has been tested against *Vibrio*, and they serve only as mechanistic precedents.

Two limitations run through this literature and constrain what can be claimed for the class. The first is provenance: no endolysin reported to act on *Vibrio* has been shown to originate from a prophage, and the best-characterized examples come from confirmed virulent phages, so endolysins support the phage-derived argument but not the prophage-specific one. The second is that intrinsic activity against intact Gram-negative cells is the exception. Lysqdvp001 requires a partner; LysMD30 has no standalone bactericidal activity; and the two non-*Vibrio enzymes* tested in seawater failed to synergize with either permeabilizer tested [56,61,62]. Reported potency is therefore a property of a formulation rather than of an enzyme, which has direct consequences for how efficacy should be specified and compared. Assay heterogeneity compounds the problem: results are reported variously as percentage biomass removal by crystal violet, log reduction in viable counts, and inhibition of biofilm formation, and these are not interconvertible. Three deliverables follow for the field, namely a defined potency unit for marine endolysins, side-by-side testing of candidate enzymes in the same seawater matrix against the same strain panel, and systematic reporting of the parent phage and its lifestyle so that provenance can be assessed at source.

### 4.2. Polysaccharide Depolymerases

Phage-encoded polysaccharide depolymerases are typically tail-spike proteins (TSPs) that recognize and specifically cleave the repeating units of capsular polysaccharide (CPS), exopolysaccharide (EPS), or lipopolysaccharide (LPS) of the host, granting access to the cell surface for DNA injection [69]. Originally described in the Podoviridae, related enzymes have since been found in the Ackermannviridae, Myoviridae, and Siphoviridae and are now applied as broadly applicable antibiofilm agents in their own right [69]. Early proof of principle came from phage SF153b, whose polysaccharide depolymerase degraded the exopolysaccharide of *Pantoea agglomerans* (formerly *Enterobacter agglomerans*) biofilms and enabled subsequent lysis by the parental phage [70]. Marine phages that infect the EPS producer *Cobetia marina* similarly encode constitutive, endo-acting polysaccharidases whose action accelerates phage adsorption under high-EPS conditions [71].

For *Vibrio*, the resource is large, but its usable fraction is smaller than the headline figures suggest, and it has barely been exploited. Bioinformatic mining showed that 79.4% of cultured and 46.2% of uncultured *Vibrio* phages carry putative depolymerase genes, although only 30.7% (66 of 215) of the candidates from cultured genomes contain a recognizable catalytic or structural domain, so most of the resource is predicted rather than annotated [18]. Biochemical validation of one candidate, Dep193, demonstrated efficient degradation of *Vibrio surface* polysaccharides and potent standalone antibiofilm activity, with synergistic bacterial clearance and delayed resistance when combined with its parental phage across multiple *Vibrio* species [18]. Two qualifications are associated with this result. Dep193 is encoded by VnaP, a novel lytic phage isolated from coastal water, so it is lytic-phage-derived rather than prophage-derived; and its primary host is *V. natriegens*, a fast-growing marine bacterium used as a laboratory chassis rather than a recognized aquaculture pathogen, with activity against *V. parahaemolyticus* and *V. alginolyticus* demonstrated as part of the broader host range. At the phage level, two novel lytic phages targeting multidrug-resistant *V. parahaemolyticus* (VpT and VpR) degraded preformed biofilms of eight non-host *Vibrio* species and complex multispecies communities [72]. The authors attribute this host-range-independent antibiofilm activity most plausibly to EPS-degrading enzymes, but no such enzyme was isolated, expressed, or assayed, so the mechanism is hypothetical and the finding belongs at the level of the phage rather than the molecule. Taken at face value, these findings indicate that exopolysaccharide matrix disruption and enhanced antimicrobial penetration, a bottleneck for antibiotic and endolysin therapy in Gram-negatives, could be addressed by exploiting the *Vibrio* phage depolymerase repertoire [73], but the supporting evidence currently consists of one validated enzyme on a non-pathogenic host.

The critical question for this class is specificity. Depolymerases recognize defined polysaccharide repeat units, so a given enzyme is expected to act on a narrow range of capsule or exopolysaccharide types, and the breadth reported for Dep193 is unusual enough to require independent confirmation on a defined panel of aquaculture isolates. In contrast, depolymerases have a property that endolysins lack: because they act on the cell surface, they are not blocked by the outer membrane, so they require no permeabilizer or engineering to reach their substrate. That makes them, in principle, the better-suited class for marine application, and the gap between that expectation and a single validated enzyme is the main open question for this class. Systematic expression and screening of the predicted depolymerase repertoire, prioritizing candidates from prophages of aquaculture-relevant pathogens rather than from cultured virulent isolates, would test the prophage-specific hypothesis directly.

### 4.3. DNases and Other Matrix-Degrading Enzymes

No phage-encoded nuclease has been characterized against a *Vibrio* biofilm. Extracellular DNA (eDNA) released by lysis or active secretion is a core structural adhesin of biofilm matrices, and enzymatic degradation of eDNA weakens biofilm integrity, disperses established biofilms, and re-sensitizes embedded cells to antimicrobials [74,75]. The proof of principle rests on enzymes that are not phage-derived: exogenous bovine DNase I removes up to 90% of *P. aeruginosa* biofilm biomass within minutes when combined with Mg^2+^ [76], and the secreted bacterial nuclease NucB from marine *Bacillus licheniformis* rapidly disperses Gram-positive and Gram-negative biofilms [77]. Recent reviews propose phage-encoded DNases as an underexploited antibiofilm class [78], and some phage endolysins, including LysAm24 and LysECD7, exhibit DNA-binding activity that contributes to matrix disruption alongside their peptidoglycan-hydrolytic function [68], but these are comparative examples from other genera. Whether dual matrix and cell-wall targeting would prove valuable against *Vibrio* biofilms, whose matrix contains substantial eDNA in addition to Vps polysaccharide, remains a testable hypothesis rather than a demonstrated result. Mining the sequenced *Vibrio* prophage catalog for nuclease domains and expressing the hits is the obvious first step, yet it has not been reported.

### 4.4. Holins and Spanins

In tailed phages of the class Caudoviricetes infecting Gram-negative hosts, lysis proceeds through three sequential steps corresponding to the three cell-envelope layers: an inner-membrane holin (or pinholin) permeabilizes the cytoplasmic membrane at a genetically scheduled time; an endolysin then reaches and degrades the periplasmic peptidoglycan; finally, a spanin complex disrupts the outer membrane, without which the cell is trapped as a non-lysed sphere [79,80]. Most phages encode a two-component spanin (an outer-membrane lipoprotein (o-spanin) plus an inner-membrane protein (i-spanin) that forms a coiled-coil periplasmic bridge) while others encode single-polypeptide u-spanins that span both membranes [80,81]. Genetic and imaging studies of the λ Rz/Rz1 system confirmed that spanin activity is essential and probably operates by catalyzing inner and outer membrane fusion, coupled to peptidoglycan degradation by the preceding endolysin step [82]. Because λ is a temperate phage, this lysis cassette is prophage-derived, making it the best-defined mechanistic precedent available for the class; it is nonetheless a comparative example, since none of this work was performed in *Vibrio*.

For applications, the interest lies less in delivering spanins alone (they are anchored in the membranes and act across the periplasm) than in exploiting the full lysis cassette. Recent reviews note that holins and spanins can be co-delivered with endolysins or expressed intracellularly by engineered phages to overcome the OM barrier that has limited the efficacy of standalone Gram-negative endolysins, and that their specific, controlled activity makes them candidates for next-generation antibacterials and drug-delivery platforms [83]. No holin or spanin from a *Vibrio* prophage has been functionally characterized. The nearest available evidence comes from a virulent phage: the *V. alginolyticus* podovirus Vp670 encodes a two-component lysis module in which *holA* is predicted to encode a holin and *cwlQ* an endolysin, with both genes proposed to have important roles in the release of phage progeny [84]. That single study defines the state of the art for *Vibrio lysis* proteins and simultaneously shows how far the prophage compartment remains unexplored. The abundant prophage catalog described in Section 2.2 is a plausible starting point for discovery, but no holin has yet been mined from it, so this class remains a research program rather than a translational option.

### 4.5. Tailocins and Phage Tail-like Bacteriocins

Tailocins (phage tail-like bacteriocins (PTLBs)) are high-molecular-weight protein complexes derived from prophages that have lost head structures and DNA, and they are the only class considered here that is prophage-derived by definition [85]. They come in two archetypes: contractile R-type particles related to P2- or T-even-like tails, and non-contractile F-type particles related to λ- or TP901-like tails; both bind bacterial surface receptors via tail fibers and kill by depolarizing the target membrane in a single, non-replicative hit [85,86,87]. Because PTLBs carry no nucleic acid, they cannot replicate, transduce, or generate the resistance dynamics that limit standard phage therapy, and their target range is defined by the tail-fiber receptor, often a specific LPS O-antigen or capsule type [88].

The most therapeutically developed PTLBs are the R- and F-type pyocins of *Pseudomonas aeruginosa*, which show sub-microgram efficacy in murine peritonitis models and can be retargeted to new species by swapping tail fibers from heterologous phages, producing engineered particles active against *E. coli* and *Yersinia pestis* [85,89]. Parallel classes have been described outside pseudomonads, including *Listeria monocins* (a new F-type family related to TP901-like tails), *Pantoea pantailocins*, and diverse F-type structures across cystic fibrosis *P. aeruginosa* collections, indicating that phage-derived bacteriocins have coevolved with multiple bacterial lineages in parallel [88,90]. All of this work is comparative: no *Vibrio* tailocin has been described, isolated, or assayed, and the class therefore contributes nothing to the current anti-*Vibrio* evidence base. This disparity illustrates the argument made in Section 1.1. The one class that is unambiguously prophage-derived is also the one class with no *Vibrio* data, while the classes with *Vibrio* data come predominantly from virulent phages. Widespread carriage of myovirus and siphovirus prophages in *V. harveyi*, *V. anguillarum*, *V. parahaemolyticus*, and *V. campbellii* (Section 2.2) makes systematic mining feasible, and the retargeting toolkit developed for R-pyocins would provide a template once a *Vibrio particle* exists to retarget, but that first step has not been taken.

### 4.6. Anti-Repressors, Small Peptides, and Phage-Derived Regulators

Beyond the lytic and matrix-degrading enzymes, prophages encode regulatory proteins and small RNAs that shape *Vibrio* pathogenesis and can themselves be repurposed as anti-virulence leads. This is the only class in which experimentally validated prophage-derived molecules are currently available, and it is therefore the compartment that carries the prophage-specific argument set out in Section 1.1.

(1). Anti-repressors are the switches that end lysogeny. In vibriophage VP882, a temperate phage, a phage-encoded VqmA homolog binds the quorum-sensing autoinducer DPO and, together with the antirepressor Qtip, sequesters the phage repressor to launch the lytic program; the same VqmA also activates the host VqmR regulon, giving the phage a lever on host QS [40]. Reprogramming VP882-derived VqmA/Qtip modules produces phages that lyse on user-defined cues, a design principle applicable to autoinducer-driven anti-*Vibrio* biocontrol [40]. Natural silencing of QS at the luxO level in some *V. parahaemolyticus* strains, conversely, blocks VP882 lysis, confirming that the host QS state directly gates phage-driven cell killing [91]. A second, functionally distinct antirepressor is available in the *V. cholerae* system and has attracted less attention as a therapeutic lead than it merits. RstC, encoded by the RS1 satellite phage, drives *recA*-dependent excision and elimination of CTXΦ from the host chromosome when delivered either by RS1φ superinfection or as additional cloned copies, generating non-toxigenic derivatives at high frequency [92]. RstC is, on the evidence currently available, the clearest example of an experimentally validated prophage-derived molecule that produces a measured anti-virulence outcome in a *Vibrio*, and its application is developed in Section 6.4. Comparable modules exist outside the genus and are cited here as comparative examples: the temperate *Pseudomonas* phage DMS3 encodes Aqs1, a 69-residue protein that binds and inhibits the LasR master QS regulator [93], and the *Aeromonas* prophage ARM81ld encodes LuxR^ARM81ld, a prophage-borne LuxR-type receptor that detects host-produced C4-HSL and is antagonized by non-cognate homoserine lactones and by a synthetic small-molecule inhibitor [94,95].

(2). Phage-encoded small RNAs (sRNAs) are an emerging regulatory class, and their direction of action requires care. Vibriophage VP882 encodes an Hfq-binding sRNA, VpdS, which promotes phage replication by tuning host and phage mRNA levels, while host-encoded sRNAs antagonize phage replication [96]. VpdS is therefore experimentally validated as prophage-derived, but it is a phage fitness factor rather than an antibacterial agent, and it is listed in Table 2 on that basis rather than as a molecule with demonstrated anti-*Vibrio* activity. The converse case is CisR in *V. cholerae*, an sRNA processed from the 3′ untranslated region of the host gene prtV, which base-pairs with the CTXΦ cep mRNA via Hfq and represses cholera-toxin phage production under stress, linking QS (via HapR) and carbon metabolism (via CRP) to CTXΦ activity [97]. CisR is host-encoded and prophage-targeting, not prophage-derived, and it acts as a host factor controlling prophage activity rather than as a member of the therapeutic class. The reciprocal sRNA regulation between phage and host nonetheless identifies candidate targets for altering *Vibrio* virulence gene expression.

These regulatory elements extend the potential repertoire beyond enzymatic killing to include precise interference with the circuits that govern *Vibrio* virulence and biofilm state, and they are the one place where the prophage compartment supplies something that virulent phages do not. Their translational development is nonetheless at a far earlier stage than that of endolysins and depolymerases, and the distance should not be understated. VqmA/Qtip is a characterized lysis-lysogeny switch rather than a formulated agent; no dosing or delivery study exists for any of these modules, and their proposed use as dispersal triggers (Section 5.4) is a design hypothesis supported by mechanism rather than by an antibacterial result. RstC is the exception in having a measured phenotypic endpoint, prophage curing, although it was demonstrated as a genetic manipulation rather than as an applied treatment. Realistically, the near-term value of this class lies in target identification and in scaffold design for QS inhibitors, with LuxR-family regulators in vibrios as the obvious target set, rather than in direct deployment.

**Table 2:** Reported molecules with antibiofilm or anti-virulence activity relevant to *Vibrio*, grouped by provenance. Block A lists experimentally validated prophage-derived molecules; block B lists molecules obtained from *Vibrio* phages that are virulent or whose lifestyle is undetermined; block C lists comparative examples from outside the genus, cited as mechanistic precedent only and not tested against *Vibrio*. Provenance categories are defined in Section 1.2.

Molecule	Source Phage and Provenance	Target *Vibrio*	Assay	Effect	Reference
**A.** Experimentally validated prophage-derived molecules (source element demonstrated to reside in a bacterial genome as a prophage or satellite prophage)					
VqmA/Qtip antirepressor pair (regulatory prophage-derived)	Vibriophage VP882 (temperate); experimentally validated prophage-derived	*V. cholerae*, *V. parahaemolyticus*	Autoinducer (DPO)-triggered lytic switch; VqmR regulon activation	DPO-driven phage-encoded VqmA, together with Qtip, sequesters phage repressor to launch lytic program and activates host VqmR regulon; reprogrammable to produce phages that lyse on user-defined QS cues	[40]
VpdS Hfq-binding sRNA (phage-encoded)	Vibriophage VP882 (temperate); experimentally validated prophage-derived	*V. parahaemolyticus*	sRNA/mRNA regulation; bidirectional phage/host sRNA network	Promotes phage replication by tuning host and phage mRNA levels; host sRNAs antagonize phage replication. Listed as a validated prophage-derived regulatory molecule, not as an agent with demonstrated anti-*Vibrio activity*	[96]
RstC antirepressor	RS1 satellite phage of *V. cholerae*; experimentally validated prophage-derived	*V. cholerae*	RS1φ superinfection or extra cloned copies; *recA*-dependent CTXΦ excision assays	Drives excision and elimination of the CTXΦ prophage from the *V. cholerae* chromosome at high frequency, generating non-toxigenic derivatives; the clearest measured anti-virulence outcome from a validated prophage-derived molecule in a *Vibrio*	[92]
**B.** Molecules from *Vibrio* phages of virulent or undetermined lifestyle (lytic-phage-derived unless stated)					
Lysqdvp001 (endolysin)	*Vibrio* phage qdvp001; lytic-phage-derived (qdvp001 is a lytic broad-spectrum myovirus, 134,742 bp, no identifiable holin gene) [7,57]	*V. parahaemolyticus*	Recombinant enzyme; PG hydrolysis; broader lytic spectrum than parental phage	Hydrolyses *V. parahaemolyticus* peptidoglycan; broader host range than qdvp001 phage	[57]
Plychap001 (truncated catalytic domain of Lysqdvp001)	*Vibrio* phage qdvp001; lytic-phage-derived (engineered truncation)	*V. parahaemolyticus*	In vitro biofilm on polystyrene; enzyme stability across temperature/pH/NaCl	Stable across broad temperature, pH, and NaCl; synergizes with EDTA; efficiently eliminates *V. parahaemolyticus* biofilms on polystyrene	[58]
Lysqdvp001-15aa (cationic peptide chimera)	*Vibrio* phage qdvp001; lytic-phage-derived (engineered cationic chimera)	*V. parahaemolyticus*	MIC/MBC; preformed biofilm removal; biofilm inhibition	MIC/MBC 0.2/0.4 mg mL^−1^; ~50% preformed biofilm removal; >90% biofilm-formation inhibition	[59]
sTHA-Lys (virtual-screening-designed artilysin)	Lysqdvp001 scaffold; lytic-phage-derived (engineered artilysin)	*V. parahaemolyticus*	Mature-biofilm removal; biofilm-formation assays	Removes >82% of mature biofilms; completely inhibits new biofilm assembly	[60]
Lysqdvp001 + ε-poly-lysine (combination)	*Vibrio* phage qdvp001 +plus food-grade AMP; lytic-phage-derived combination	*V. parahaemolyticus*	Cod, penaeid shrimp, oyster tissue matrices; biofilms on polystyrene, glass, stainless steel	3.75, 4.16 and 2.50 log_10_ CFU g^−1^ reductions in cod, shrimp and oyster tissue respectively (range 2.50 to 4.16); 44 to 68% biofilm removal on polystyrene, glass and stainless steel; unmodified Lysqdvp001 alone had little effect on preformed biofilm	[61]
LysMD30 (L-alanyl-D-glutamate peptidase; genome-mined)	Genome-mined from 163 *Vibrio* phages; parent phage not named and lifestyle not reported; provenance undetermined	*V. parahaemolyticus*, *V. harveyi*, *V. alginolyticus*	Checkerboard with colistin; biofilms on polystyrene and stainless steel; *Galleria mellonella* infection	Synergy with colistin (FICI < 0.5); 4-fold reduction in colistin MIC; >3 log reduction in biofilm viable counts within 1 h on polystyrene and stainless steel; 100% *Galleria* protection; delays colistin resistance emergence. Colistin is a WHO highest-priority critically important antimicrobial (Section 8.2)	[62]
LysVPB (17 kDa Ca^2+^-boosted endolysin)	Unnamed *V. parahaemolyticus*-infecting phage; provenance undetermined	*V. parahaemolyticus*	Recombinant enzyme; biochemical characterization; modular domain analysis	Unusually divergent, calcium-boosted endolysin; modular architecture supports domain swapping for chimeric design	[65]
LysVpKK5 (amidase_2 endolysin)	Unnamed marine vibriophage; provenance undetermined	*V. parahaemolyticus*	Enzyme biochemistry in seawater: pH profile	Retains ~50% activity in seawater; prefers alkaline pH	[66]
Vplys60 (*Pichia*-expressed endolysin)	*V. parahaemolyticus* phage qdv001 (same endolysin lineage as qdvp001); lytic-phage-derived	*V. parahaemolyticus*	Recombinant *Pichia pastoris* X-33 whole-cell biocatalyst; in vitro biofilm; in vivo *Artemia franciscana*	91.6% biofilm inhibition at 75 μg mL^−1^; strong amidase activity; enhances *Artemia* survival with reduced *Vibrio* load in vivo	[98]
LysVPp1 (phage-derived lysin)	Endolysin of an unnamed *V. parahaemolyticus* phage; applied alongside, but not derived from, the lytic phage CH20; provenance undetermined	*V. alginolyticus*, *V. parahaemolyticus*, *V. splendidus*	Rotifer and fish-larvae bioassays; microbiota impact via 16S	Significant reduction in *Vibrio* load in rotifers and fish larvae; limited off-target impact on rearing microbiota	[99]
Dep193 (polysaccharide depolymerase)	*Vibrio* phage VnaP; lytic-phage-derived (VnaP is a novel lytic phage isolated from coastal water)	*V. natriegens* AbY-1805 (primary host; not an aquaculture pathogen), *V. parahaemolyticus*, *V. alginolyticus*, *V. diabolicus*, *V. atypicus*	Enzymatic degradation of surface polysaccharide; standalone biofilm assays; combination with parental phage	Efficient degradation of *Vibrio* surface polysaccharides; potent standalone antibiofilm activity; synergistic bacterial clearance with parental phage; delayed resistance emergence across multiple *Vibrio* species	[18]
Phage JSF7 biofilm-dispersing factor (predicted polysaccharide-degrading open reading frame; assayed as crude recombinant extract)	*V. cholerae* O1-specific phage JSF7; no lysogeny reported; provenance undetermined	*V. cholerae* (plus cross-genus activity)	Cloned into *E. coli*; crude recombinant extract on biofilms of *E. coli*, *Shigella*, *Pseudomonas*, *V. cholerae*	Crude recombinant extract disperses *V. cholerae* and cross-genus biofilms, and improves recovery of *V. cholerae* O1 from environmental water by enrichment culture; demonstrated application is bacteriological detection, not water treatment	[100]
Biofilm-degrading activity of phages VpT and VpR whole phages; responsible enzyme not isolated, mechanism hypothetical)	Novel lytic *Vibrio* phages VpT and VpR; lytic-phage-derived	*V. parahaemolyticus* (host); 8 non-host *Vibrio* species (cross-species biofilm activity)	Preformed biofilm degradation across single-species and multispecies *Vibrio* communities	Degrades preformed biofilms of 8 non-host *Vibrio* species and complex multispecies *Vibrio* communities; host-range-independent antibiofilm activity	[72]
**C.** Comparative examples from outside the genus *Vibrio* (mechanistic precedent only; not tested against *Vibrio*)					
KZ144 and LysPA26 endolysins tested in natural seawater (comparative example)	*Pseudomonas aeruginosa* phages φKZ and a *P. aeruginosa* phage; lytic-phage-derived, non-*Vibrio enzymes*	*V. parahaemolyticus*	Muralytic activity in natural seawater; EDTA and oregano essential oil as permeabilizers	Both endolysins retained muralytic activity in seawater, but neither synergized with EDTA or oregano essential oil, and the applied protein was metabolized by the pathogen as a growth substrate. Negative result	[56]
LysAm24, LysAp22 and LysECD7 (dual PG hydrolase +plus matrix-binding activity; comparative example)	*Acinetobacter* phages AM24 and Ap22 and *E. coli* phage ECD7; lytic-phage-derived, non-*Vibrio enzymes*	Gram-negative biofilms; not tested against *Vibrio*	eDNA binding; peptidoglycan hydrolysis; matrix disruption	LysAm24 binds extracellular DNA, LysAp22 disrupts acidic exopolysaccharides, and LysECD7 combines both activities with peptidoglycan hydrolysis; proposed, but not tested, for *Vibrio* biofilms whose matrix contains eDNA alongside Vps polysaccharide	[68]
Aqs1 (69-residue anti-activator; comparative example)	*Pseudomonas* phage DMS3 (temperate); prophage-derived, non-*Vibrio*	Not tested against *Vibrio*	Binding and inhibition assays against the LasR master quorum-sensing regulator	Small prophage-encoded polypeptide acts as a direct QS-inhibitor scaffold; proposed as a design template for LuxR-family regulators in vibrios	[93]
LuxR^ARM81ld quorum-sensing receptor (comparative example)	*Aeromonas* prophage ARM81ld (temperate); prophage-derived, non-*Vibrio*	Not tested against *Vibrio*	Autoinducer binding and lysis reporter assays; antagonism by non-cognate homoserine lactones and a synthetic inhibitor	Prophage-encoded LuxR-type receptor detects host-produced C4-HSL and activates lysis, and is antagonized by C8-HSL and by a small-molecule inhibitor, showing that mixed-community QS signals can be used to modulate prophage behavior	[94,95]
R-type and F-type tailocins (pyocins, monocins, pantailocins; comparative example)	Domesticated defective prophages; prophage-derived by definition, non-*Vibrio*	No *Vibrio tailocin* has been described	Murine peritonitis model; tail-fiber retargeting; receptor-binding assays	Sub-microgram efficacy against *P. aeruginosa* and retargetable to *E. coli* and *Yersinia pestis*; the only class considered here that is exclusively prophage-derived, and the only class with no *Vibrio data*	[85,89,90]

Note: The *V. cholerae* small RNA CisR, processed from the 3′ untranslated region of the host gene *prtV*, represses production of the cholera-toxin phage CTXΦ through Hfq-dependent base-pairing with the *cep* transcript [97]. It is host-encoded and prophage-targeting rather than prophage-derived, and it is therefore not listed above; it is discussed in Section 4.6 and Section 6.4 as a host factor that controls prophage activity. Host mucin-derived O-glycans, which block CTXΦ toxigenic conversion at the epithelial surface [101], belong to the same category. The prophage-encoded fitness genes *VpaChn25_0734* and *VpaChn25_0724* of *V. parahaemolyticus* CHN25 [41,102] are prophage-derived targets rather than candidate agents and are discussed in Section 3.2 and Section 6.2.

## 5. Prophage-Derived Strategies for Biofilm Control in *Vibrio*

### 5.1. Biofilm Formation and Its Role in Vibriosis

**Figure 1:**
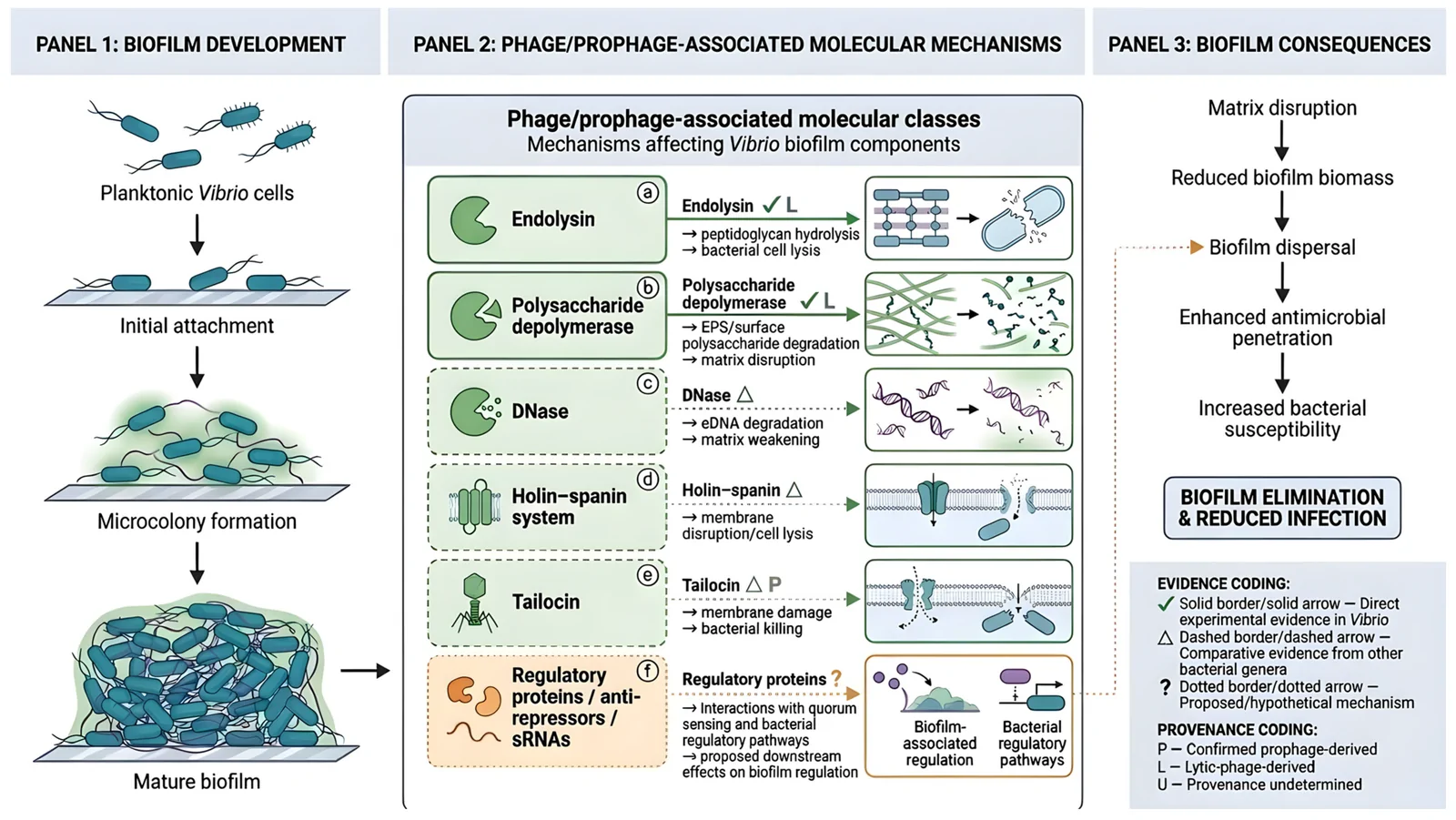
Established, comparative, and proposed mechanisms of phage- and prophage-derived biofilm disruption in *Vibrio*. Evidence status and molecular provenance are indicated separately. ✓ Solid indicates direct experimental evidence in *Vibrio*, △ dashed indicates comparative experimental evidence from other bacterial genera, and ? dotted indicates a proposed or hypothetical mechanism. P, L, and U denote confirmed prophage-derived provenance, lytic phage-derived provenance, and undetermined provenance, respectively. (**a**) Endolysin-mediated peptidoglycan hydrolysis and bacterial cell lysis have been experimentally demonstrated against *Vibrio*, although the characterized examples shown here are derived from lytic phages [57,58,59,60,61,62,65,66]. (**b**) Polysaccharide depolymerases, including Dep193, have demonstrated activity against *Vibrio* surface polysaccharides and biofilms, with the characterized examples originating from lytic phages [18]. (**c**) Nuclease-mediated eDNA degradation has experimental support in biofilms of other bacterial genera, but a phage-encoded nuclease has not been experimentally validated for controlling *Vibrio* biofilms [74,75,76,77,78]. (**d**) Holin and spanin-mediated membrane disruption and cell lysis are established phage mechanisms, but no holin or spanin from a *Vibrio* prophage has been functionally characterized for biofilm control [79,80,81,82,83]. (**e**) Tailocins are prophage-derived bacteriocins with experimentally demonstrated antibacterial activity in other bacterial genera, but no *Vibrio* tailocin has yet been experimentally characterized for this application [85,86,87,88,89,90]. (**f**) Phage- and prophage-encoded regulatory proteins, anti-repressors, and small regulatory RNAs can interact with quorum-sensing and bacterial regulatory pathways, but their downstream use for direct control of *Vibrio* biofilms remains proposed and requires experimental validation [40,94,95,96]. Direct experimental activity against *Vibrio* does not establish prophage provenance; evidence status and molecular origin are therefore shown independently.

Section 1 and Section 3.3 established that biofilms are central to *Vibrio* persistence and virulence. In aquaculture, this general fact translates into concrete disease pressure. Biofilm-forming vibrios are among the most common aquatic pathogens, and their embedded lifestyle drives both the economic losses of vibriosis and the accelerated selection of antibiotic-resistant strains that follows repeated prophylactic dosing [103]. Signs of *Vibrio* disease in penaeid shrimp (lethargy, appendage necrosis, luminescence, empty midgut, mass larval mortality) are closely linked to biofilm formation and quorum-sensing coordination, so disrupting these two networks has emerged as a management strategy to avoid the resistance treadmill of simple bactericides [104]. Figure 1 depicts the principal mechanisms by which prophage-derived molecules disrupt *Vibrio* biofilms, from attachment through matrix degradation to dispersal.

The problem is worsened by biofilm colonization of hatchery infrastructure. *V. parahaemolyticus* readily forms biofilms on high-density polyethylene, stainless steel, glass, and shellfish surfaces, and is not eliminated from these matrices even at 20 ppm sodium hypochlorite [105]; *V. campbellii* colonizes tile and plastic surfaces within 48 to 96 h, indicating that standard hatchery construction materials themselves act as reservoirs [106]. At the seafood-industry scale, *V. parahaemolyticus* biofilms confer greater stress resistance, aid persistence, and threaten global supply as warming expands its geographic range [107]. These constraints motivate biofilm-targeted interventions (prevention of attachment, matrix degradation, active dispersal, and adjuvant synergy) that phage-derived molecules can in principle deliver.

Whole lytic phages and purified phage-derived proteins represent different intervention formats. Whole phages can replicate within susceptible bacterial populations, but their activity depends on host recognition and may select for phage-resistant populations. Purified proteins have a defined composition and do not replicate or mediate transduction, but they require external dosing and may be limited by protein stability or access to the bacterial target. Whole phage studies are therefore discussed in this section as comparative evidence rather than as direct evidence for prophage-derived products.

### 5.2. Prevention of Initial Surface Attachment

The clearest translational role in *Vibrio* biofilm control is prophylaxis: intercepting the pathogen in planktonic form or during early attachment, before a mature matrix accumulates. Almost all of the evidence in this subsection comes from whole virulent phages rather than from prophage-derived molecules, and it provides the comparator against which prophage-derived options must be judged, not as evidence for them. Screening of 34 *V. parahaemolyticus*-specific phages showed that individual phages and cocktails effectively prevent biofilm formation but fail to eradicate established biofilms, framing early intervention as the highest-value window [108]. Applied prophylactically in an artificial contamination model, phage pVp-1 reduced *V. parahaemolyticus* in live oysters from 8.9 × 10^6^ to 1.4 × 10^1^ CFU mL^−1^ within 72 h of bath immersion after challenge, close to a six log_10_ reduction, a magnitude that is not achievable once the pathogen has established mature aggregates [109].

At the hatchery front end, phage cocktails can pre-treat the live feeds that carry vibrios into larval systems. The φSt2/φGrn1 cocktail, applied at MOI = 100 to *Artemia salina* cultures, achieved a 93% reduction in *V. alginolyticus* within 4 h, effectively decontaminating the feed before it is offered to seabream larvae [110]. In a simulated shrimp-hatchery microcosm, phage VHP6b (broadly infective against 27 *V. harveyi* strains) improved postlarval survival by 40 to 60% under conditions designed to mimic real hatchery water chemistry, directly demonstrating that pre-attachment phage delivery averts luminous vibriosis [111]. New broad-host phage isolates continue to expand this pipeline: siphovirus R23Z targets 16 *Vibrio* strains across four species and raises shrimp survival by >16% [112], while two podoviruses (vB_VcaP_R24D and R25D) show clean lytic profiles and stable activity across 4 to 45 °C and pH 3 to 10, matching typical hatchery water conditions [113]. Prophage-derived endolysins can play the same prophylactic role, and LysVPp1 applied to rotifers and fish larvae reduces *V. alginolyticus*, *V. parahaemolyticus*, and *V. splendidus* loads without perturbing the wider microbiota, providing an antibiotic-free biosecurity layer during the critical live-feed-to-larvae transfer [99,114].

### 5.3. Biofilm Matrix Degradation

Once a *Vibrio* biofilm has matured, the matrix itself must be dismantled before antimicrobials can reach the embedded cells. This is where the enzymatic classes introduced in Section 4.2 and Section 4.3 become central in principle, although only the depolymerases currently have any evidence from *Vibrio*. The first biochemically validated example, Dep193 from the lytic phage VnaP, efficiently degrades *Vibrio* surface polysaccharides, exhibits potent standalone antibiofilm activity, and synergizes with its parental phage to clear multiple *Vibrio* species while delaying the emergence of resistance, with the qualifications on host species and provenance set out in Section 4.2 [18]. A conceptually similar experiment in the *V. cholerae* system cloned a predicted polysaccharide-degrading gene from phage JSF7 into *E. coli* and used the crude recombinant extract to disperse *E. coli*, *Shigella*, *Pseudomonas*, and *V. cholerae* biofilms, and supplementing enrichment medium with the extract improved recovery of *V. cholerae* O1 from environmental water [100]. Two limits apply: the enzyme is predicted rather than purified and characterized, and the demonstrated application is bacteriological detection rather than water treatment, so extension to the latter is at present hypothetical. Beyond monovalent enzymes, whole lytic phages carrying presumptive EPS-degrading activity disrupt biofilms outside their host range: the *V. parahaemolyticus* phages VpT and VpR degrade preformed biofilms of eight non-host *Vibrio* species and complex multispecies communities, which makes them practical polyvalent antibiofilm agents for aquaculture systems even though the responsible enzyme has not been identified [72].

The mechanism by which matrix degradation improves outcomes is well established outside *Vibrio*: an *Aeromonas punctata*-derived capsular depolymerase, when applied with gentamicin, dispersed the *K. pneumoniae* biofilm matrix and allowed the antibiotic to reach previously protected cells, producing significant additional killing that neither agent alone achieved [115]. Reviews of phage depolymerases as biofilm-control agents converge on the same principle: depolymerases degrade the matrix, remove the physical barrier to antibiotic penetration, and expose sessile cells to killing by antibiotics, immune effectors, or the phage itself [116]. For *Vibrio* biofilms whose matrix contains substantial eDNA in addition to Vps polysaccharide (see Section 4.3), endolysins with combined DNA-binding and PG-hydrolytic activity, such as LysAm24 and LysECD7, provide dual-target matrix disruption in a single protein, and phage-encoded DNases have been explicitly proposed as an underexploited *Vibrio* antibiofilm class [78].

### 5.4. Biofilm Dispersal Mechanisms

Prevention targets the earliest stage of the biofilm life cycle and matrix degradation targets the physical barrier, but neither returns an established, metabolically stable population to a susceptible state. Active dispersal, driving mature biofilms back to a planktonic state, is a distinct third strategy, and characterizing the native dispersal circuitry of *Vibrio* is a prerequisite for engineering any phage-derived trigger.

*V. cholerae* integrates dual sensory inputs to control dispersal: the general stress response (activated by starvation) is coupled to quorum sensing so that the cell disperses only when the biofilm is both stressed and populous, avoiding premature exit from small aggregates [117]. A genetic screen identified three effector classes downstream of that decision (signaling proteins, matrix-degrading enzymes, and motility factors), with the DbfS/DbfR two-component system as the master switch, LapG (adhesin cleavage) and RbmB (matrix-polysaccharide digestion) as the enzymatic effectors, and CheY3-mediated reorientations providing the flagellar force needed to escape the porous matrix [118]. The quorum-sensing arm itself operates as a coincidence detector integrating the intra-genus autoinducer CAI-1 with the universal AI-2, so that only simultaneous activation of both signals authorizes dispersal, a design principle that could be exploited by exogenous AI-2 or AI-2 mimics to force dispersal in a controlled way [119]. Analogous circuitry operates in *V. fischeri*, where LapG cleaves the biofilm-promoting adhesin LapV under c-di-GMP control by LapD, extending the LapG-adhesin architecture across *Vibrio* species [120].

Prophage-derived molecules could in principle plug into this circuitry, although no such construct has yet been built or tested. Phage-encoded depolymerases such as Dep193 phenocopy the RbmB matrix-digestion step from outside the cell (Section 5.3), and the VP882 VqmA/Qtip anti-repressor pair (Section 4.6) reads the same DPO autoinducer signal that the host uses to govern virulence and biofilm behavior, which identifies a mechanistic docking point for engineered dispersal-triggering constructs that would lyse or eject bacteria on QS cues [40]. This convergence between endogenous *Vibrio* dispersal control and phage-encoded signaling modules makes regulatory prophage cargo a strategic class alongside the more mature endolysin and depolymerase toolkits, though the proposal remains hypothetical and is a research direction rather than an available reagent.

### 5.5. Synergy with Antibiotics and Other Antimicrobials

For established *Vibrio* infections in commercial aquaculture, monotherapy (whether phage, endolysin, depolymerase, or antibiotic alone) usually falls short. The convergent finding from the last five years is that combinations of phage-derived molecules with conventional antimicrobials produce disproportionately larger biofilm reductions and delay resistance emergence.

In *Vibrio*, the principle is being validated rapidly. Phage Φ170 combined with doxycycline synergistically reduced multidrug-resistant *V. parahaemolyticus* loads in a zebrafish challenge model, alleviated liver and intestinal damage, and suppressed inflammation beyond what either agent achieved alone [121]. At the enzyme level, the genome-mined endolysin LysMD30 lacks intrinsic bactericidal activity but synergizes strongly with colistin against *V. parahaemolyticus*, *V. harveyi*, and *V. alginolyticus* (FICI < 0.5), reducing the colistin MIC fourfold and lowering biofilm viable counts by more than 3 logs on polystyrene and stainless steel within 1 h [62], subject to the One Health constraint on colistin set out in Section 4.1 and Section 8.2. Mechanistic detail, the non-antibiotic permeabilizer alternatives, and the documented failure of permeabilizer pairing in natural seawater [56] are covered in Section 8.2.

## 6. Prophage-Derived Strategies for Virulence Suppression

**Figure 2:**
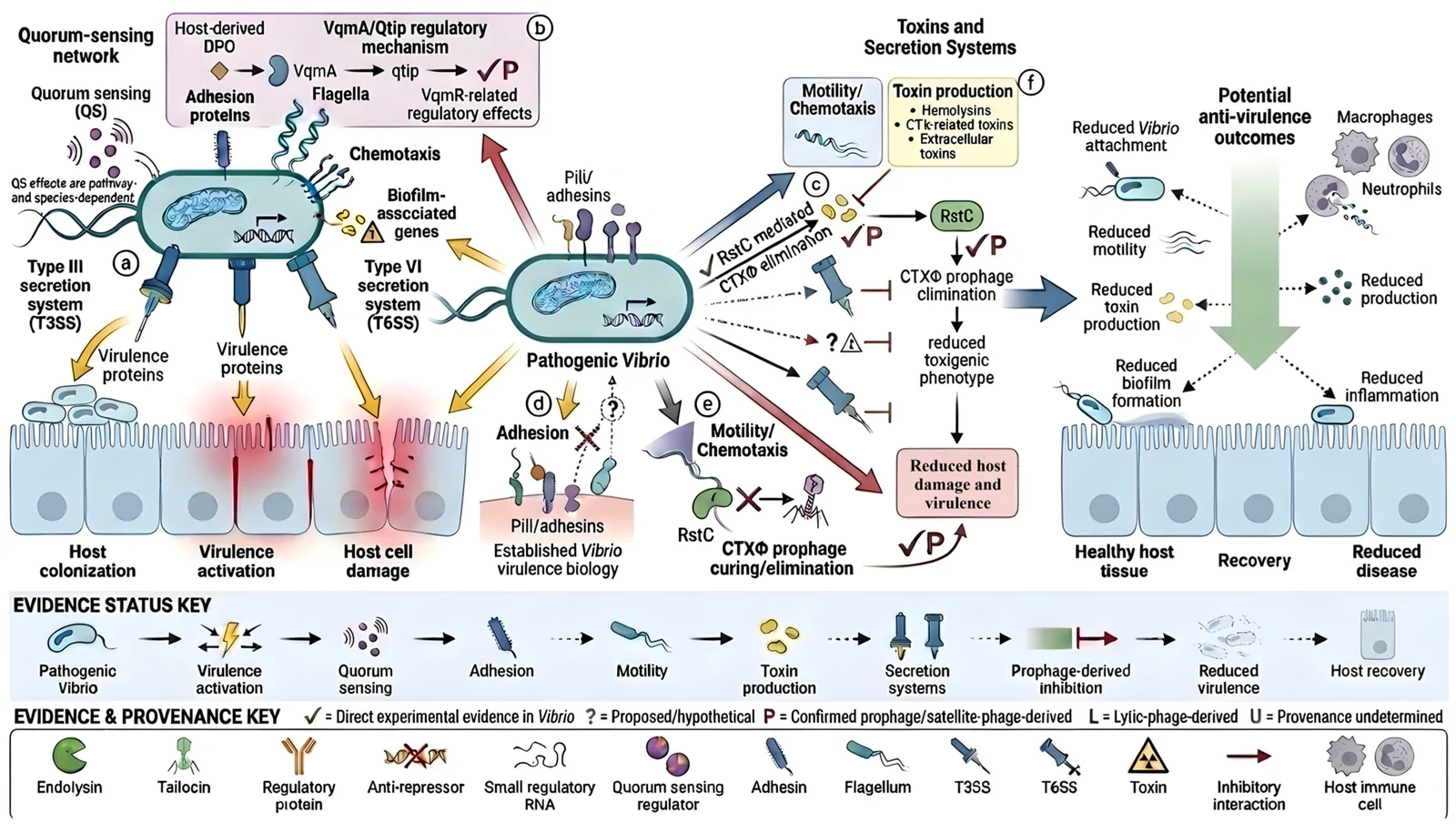
Established and proposed anti-virulence targets of prophage-derived molecules in *Vibrio*. The figure distinguishes established *Vibrio* virulence pathways from experimentally validated and proposed prophage-derived intervention points. ✓ Solid indicates direct experimental evidence in *Vibrio*, △ dashed indicates comparative evidence, and ? dotted indicates a proposed or hypothetical mechanism. P, L, and U denote confirmed prophage or satellite-phage-derived provenance, lytic phage-derived provenance, and undetermined provenance, respectively. (**a**) Quorum sensing is an established regulatory network controlling multiple *Vibrio* virulence-associated outputs, including adhesion, motility, toxin production, and secretion systems [122,126,130,133,137]. (**b**) The VP882 VqmA/Qtip system provides experimentally validated evidence for a prophage-derived regulatory interaction involving the host-derived DPO signal and the VqmR pathway [40]. This represents validated molecular regulation rather than a validated anti-virulence treatment. (**c**) The RS1-encoded antirepressor RstC has demonstrated an anti-virulence outcome in *V. cholerae* by promoting CTXΦ elimination and generating non-toxigenic derivatives [92]. (**d**) Adhesion and pili-associated attachment are established components of *Vibrio* virulence biology, but direct suppression of these processes by prophage-derived molecules remains proposed [133,137]. (**e**) Motility and chemotaxis are established virulence-associated traits in *Vibrio*, but their direct suppression by prophage-derived molecules has not been experimentally demonstrated [126]. (**f**) Toxin production and T3SS/T6SS are established components of *Vibrio* virulence, whereas direct prophage-derived suppression of these pathways remains proposed or insufficiently validated, apart from the RstC-associated CTXΦ toxin-loss phenotype described above [130,133,137]. The pathways are therefore presented as potential anti-virulence targets rather than as experimentally validated prophage-derived treatments. Quorum-sensing effects are context-dependent and may differ among *Vibrio* species and individual virulence outputs.

Killing *Vibrio* cells and dispersing their biofilms (as discussed in detail in Section 4 and Section 5) imposes strong selection for resistance. An increasingly attractive complement is to disarm the pathogen rather than eliminate it: attenuate the virulence program so the bacterium can persist harmlessly, or at least fail to establish disease. Prophage-derived molecules are well placed for this because prophage cargo is already embedded in the regulatory circuitry that vibrios themselves use to control virulence, quorum sensing, adhesion, motility, toxin production, and secretion systems. Figure 2 maps the anti-virulence targets of prophage-derived molecules, including quorum sensing, adhesion, motility, toxin production, and secretion systems.

### 6.1. Interference with Quorum Sensing

Quorum sensing (QS) is the master coordinator of *Vibrio* virulence: at low cell density, AphA and LuxO-P dominate and virulence factors are expressed, whereas at high cell density, accumulated autoinducers dephosphorylate LuxO and derepress the master regulator HapR/OpaR/LuxR, which then represses virulence and biofilm genes [122]. Because this cascade converges on a small set of LuxO- and LuxR-family regulators, it presents a discrete set of molecular targets that prophage-derived reagents can hit.

The first, and most direct, class comes from phage genomes themselves. Prophages carry homologs of bacterial LuxR-type AHL receptors that bind the same C4 to C8 acyl-homoserine lactones as their hosts, allowing phages to “listen in” on host cell-density state to time their lysis-lysogeny decisions [94]. LuxR\^ARM81ld, encoded by an *Aeromonas* prophage, detects host-produced C4-HSL and activates lysis; the same receptor is antagonized by non-cognate HSLs (e.g., C8-HSL from co-occurring *V. fischeri*) and by a synthetic small-molecule inhibitor, so mixed-community QS signals can be co-opted to modulate phage behavior and, by extension, host QS output [95]. In *Vibrio* proper, the paradigmatic example remains vibriophage VP882: its phage-encoded VqmA/Qtip anti-repressor pair reads the host QS autoinducer DPO and simultaneously activates the host VqmR regulon, giving a single phage-derived module control over both the phage lifecycle and host virulence programs (Section 4.6) [40]. Whether these receptors and antirepressor scaffolds are used as detection tools, as engineered synthetic circuits, or as leads for QS-inhibitor design, they open a rational route to virulence attenuation that killing-based strategies cannot.

A second, indirect route emerges from phage-driven perturbation of QS itself. In *V. alginolyticus*, acquisition of bacteriophage resistance downregulates the QS receptors LuxM, LuxN, and LuxP together with the downstream regulators LuxU, *aphA*, and *luxR*, and the resulting metabolic reprogramming produces reduced planktonic growth, altered biofilm formation, and (importantly) reduced virulence, an outcome that mirrors what QS inhibitors are designed to achieve [123]. Analogous logic operates from the phage side: the DMS3-encoded 69-residue protein Aqs1 in *Pseudomonas* binds and inhibits the LasR master QS regulator, demonstrating that small phage-encoded polypeptides can act as direct QS-inhibitor scaffolds, a design principle immediately transferable to LuxR-family regulators in vibrios [93].

### 6.2. Inhibition of Adhesion and Host Colonization

For *Vibrio* infections to progress, the pathogen must first reach and adhere to the host epithelium. Phage treatment can act as a colonization-blocker in vivo. In a zebrafish (*Danio rerio*) larval model, a naturalistic host for *V. cholerae*, bacteriophage ICP1 significantly reduced intestinal *V. cholerae* colonization independent of microbiome composition, with light and electron microscopy directly visualizing phage-driven clearance from the epithelium [124]. Prophage-derived molecules can operate on the same axis. Host mucin polymers themselves act as regulators of prophage-driven virulence: mucin-associated O-glycans (specifically Core 2 structures) block CTXΦ toxigenic conversion of non-toxigenic *V. cholerae* and suppress expression of both the toxin-coregulated pilus and cholera toxin by interfering with the TcpP/ToxR/ToxT pathway, illustrating how phage receptor and adhesion functions can be simultaneously neutralized at the mucosal surface [101]. Prophage-encoded gene products also modulate adhesion from the bacterial side, and two adjacent genes of the same prophage cluster in *V. parahaemolyticus* CHN25 should be distinguished. Deletion of *VpaChn25_0734* reduces swimming motility, cell-surface hydrophobicity, biofilm formation, and cytotoxicity toward human intestinal epithelial cells (Section 3.2) [41], whereas deletion of the adjacent *VpaChn25_0724* compromises cell membrane integrity and growth [102]. Both results indicate that prophage cargo can be exploited to attenuate colonization when the underlying gene is deleted or antagonized, and both identify prophage-derived targets rather than prophage-derived agents.

### 6.3. Reduction in Motility and Chemotaxis

Flagellar motility is required for *Vibrio* invasion and colonization, and its regulation is tightly integrated with QS in *V. cholerae, V. parahaemolyticus, V. harveyi*, and *V. alginolyticus*; motility and biofilm/virulence are therefore both plausible targets for a single class of intervention [125]. In *V. harveyi*, QS positively regulates flagellar motility: autoinducer-synthase mutants show markedly reduced swimming, restored by synthetic autoinducers, and expression of early, middle, and late flagellar genes is significantly lower in QS-inactive luxO(D47E) mutants than in wild-type or maximally QS-active luxO(D47A) mutants [126]. The motility inhibitor phenamil also reduces *V. harveyi* virulence toward gnotobiotic brine shrimp larvae, direct proof that motility-targeted anti-virulence works in an aquaculture host [126]. In *V. alginolyticus*, the QS enzyme LuxS controls polar and lateral flagellar biogenesis via *flaK/lafK/fliS/lafA*; deletion of *luxS* produces motility-defective, low-virulence phenotypes [127]. Phage-encoded QS interventions (Section 6.1) that shift *Vibrio* toward the high-cell-density QS state can therefore indirectly reduce motility, and prophage-encoded regulators, such as *VpaChn25_0724*, directly compromise swimming when disrupted; its deletion mutant is defective in growth and swimming motility at low temperatures relevant to hatchery conditions [102].

### 6.4. Suppression of Toxin Production

Toxin production in vibrios is often prophage-driven, so prophage-derived reagents can be deployed to interrupt toxin conversion or expression rather than to clear cells alone. The CTXΦ paradigm remains central. Naturally occurring toxigenic *V. cholerae* strains are inducible lysogens: mitomycin C converts ~34% of clinical/environmental isolates into CTXΦ-producing lysogens, and induced particles can transmit CTXΦ to non-toxigenic recipients in the infant-mouse intestine, illustrating that induction is a route by which phage-derived cargo drives toxin dissemination [128]. Interrupting this axis is therefore an anti-toxin strategy in its own right.

Several validated routes exist. Spontaneous loss of the CTXΦ prophage in *V. cholerae* El Tor produces isogenic non-toxigenic mutants with simultaneously reduced colonization ability, soluble haemagglutinin/protease and thermolabile hemolysin production, and motility, showing that prophage curing broadly attenuates virulence rather than only removing toxin [129]. The satellite phage RS1 provides a molecular tool for this: the RS1-encoded antirepressor RstC, delivered as either RS1φ superinfection or extra cloned copies, drives *recA*-dependent excision and elimination of CTXΦ from the *V. cholerae* chromosome, producing non-toxigenic derivatives at high frequency [92]. Host and phage RNAs also modulate cholera toxin gene expression post-transcriptionally: the *V. cholerae* CisR sRNA (from the *prtV* 3′UTR) base-pairs via Hfq with the CTXΦ *cep* transcript and represses CTXΦ production under stress, integrating QS (HapR) and metabolism (CRP) inputs and providing a direct RNA-based lead for toxin suppression (Section 4.6) [97]. Mucin-derived O-glycans further block CTXΦ-mediated toxigenic conversion and cholera-toxin expression at the epithelial surface (Section 6.2) [101]. These examples define a coherent anti-toxin strategy built entirely from prophage-derived components: prevent CTXΦ acquisition, drive prophage curing, and repress residual CTXΦ transcripts.

### 6.5. Modulation of Secretion Systems

The type III (T3SS) and type VI secretion systems (T6SS) are the principal delivery devices for *Vibrio* virulence effectors, and both are heavily QS-regulated, placing them within reach of QS-targeting prophage-derived reagents.

(1). T3SS *V. parahaemolyticus* requires two T3SSs plus a thermostable direct hemolysin for full virulence, with T3SS2 encoded within the 80 kb Vp-PAI on chromosome II [130]. Expression is choreographed by QS-controlled regulators AphA (low cell density) and OpaR (high cell density) together with the AraC-family regulator QsvR: AphA activates T3SS1 by binding the exsBAD-vscBCD operator at low density, OpaR replaces AphA at high density to shut T3SS1 off and induce Vp-PAI, and QsvR activates the whole set at high density [130]. Two additional layers of regulation were recently added: the TetR-family repressor TftR represses T3SS1 by enhancing OpaR production, while the LysR-family activator VltR directly activates exsBA transcription independently of AphA/OpaR, creating opposing rheostats whose imbalance attenuates or amplifies T3SS-mediated cytotoxicity [131]. T3SS2 is further gated by the secreted anti-activator VtrN, whose export in response to host–cell contact represses its own gene cluster, a natural feedback loop that anti-virulence engineering can lock in the “off” state [132]. Because AphA, OpaR, and QsvR are downstream targets of the same LuxR/LuxO cascade that phage-encoded VqmA-family regulators can manipulate (Section 6.1), a QS-targeting prophage-derived intervention would be expected to propagate to T3SS regulation, but not in a single direction: driving the high-cell-density state would attenuate T3SS1 while simultaneously inducing Vp-PAI and T3SS2 in the same organism [130]. The sign of the effect is therefore species-dependent and pathway-dependent, and the constraints this places on target selection are set out in Section 6.6.

(2). T6SS *V. parahaemolyticus* carries two T6SSs whose regulation is similarly QS- and surface-dependent: T6SS1 is active under warm, high-salt marine conditions and contributes to antibacterial competition and adhesion to epithelial cells, while T6SS2 operates at low salt and low temperature and controls adhesion, biofilm formation, motility, and macrophage-autophagy induction [133,134]. In the AHPND-causing *V. parahaemolyticus* strain 13-306/D4, inactivation of T6SS1 significantly reduces lethality against *L. vannamei* postlarvae under warm marine conditions, directly demonstrating that T6SS is an aquaculture-relevant virulence target [135]. Mechanistically, T6SS1 structural components VipA1 and Hcp1 secrete 149 T6SS1-dependent proteins, and their combined deletion attenuates virulence by 52.7%, restores biofilm hyper-formation, reduces motility, cuts mouse mortality from 91.7% to 50%, and lifts NF-κB suppression, exposing T6SS1 as a driver of immune evasion in addition to interbacterial competition [136]. QsvR provides the direct QS input into T6SS2; it binds the promoters of the three T6SS2 operons (VPA1027-1024, VPA1043-1028, VPA1044-1046), activates their transcription, and its deletion significantly reduces *V. parahaemolyticus* adhesion to HeLa cells [137].

T3SS and T6SS regulation converges on a small set of QS-dependent transcription factors (AphA, OpaR, QsvR, plus TftR/VltR and VtrN), and each of these nodes is in principle within reach of the prophage-derived QS scaffolds described in Section 4.6 and Section 6.1. That convergence is why anti-secretion-system approaches, historically pursued with chemical anti-virulence inhibitors, are increasingly plausible using prophage-encoded regulators as designer QS mimics, dominant-negative anti-repressors, and Hfq-binding sRNAs, either alone or as adjuvants alongside the killing- and biofilm-focused strategies of Section 4 and Section 5. The direction of that effect cannot be assumed, however, and Section 6.6 sets out why.

### 6.6. Target-Selection Constraints: Quorum Sensing Does Not Attenuate Virulence Uniformly

Section 6.1, Section 6.2, Section 6.3, Section 6.4 and Section 6.5 are often read as implying a single therapeutic direction: that driving *Vibrio **into*** the high-cell-density QS state attenuates virulence. The evidence reviewed above does not support that generalization, and stating the constraint explicitly is necessary before any QS-directed prophage-derived reagent is designed. The canonical cascade holds that at high cell density, accumulated autoinducers dephosphorylate LuxO, derepressing the master regulator HapR/OpaR/LuxR, which in turn represses biofilm formation and several virulence outputs [14,122]. Three of the results summarized above run in the opposite direction. In *V. parahaemolyticus*, OpaR shuts off T3SS1 at high cell density but simultaneously induces the 80 kb Vp-PAI carrying T3SS2, and the AraC-family regulator QsvR activates the set at high density [130]; QsvR also binds and activates the promoters of all three T6SS2 operons [137]; and in *V. harveyi*, QS positively regulates flagellar motility, with QS-inactive *luxO*(D47E) mutants showing reduced expression of early, middle, and late flagellar genes [126].

The practical consequence is that a single QS-directed intervention would attenuate some virulence outputs while activating others in the same organism. Target selection must therefore specify the species, the output, and the desired direction, rather than treating virulence as a unitary phenotype. Reading across the studies cited in this section, driving toward the high-cell-density state is expected to be beneficial for *V. cholerae* biofilm repression [14], for *V. parahaemolyticus* T3SS1 [130], and for the general reduction in biofilm accumulation, but counterproductive for *V. parahaemolyticus* T3SS2 and Vp-PAI expression [130], for T6SS2 [137], and for *V. harveyi* motility [126]. The opposite intervention, locking the low-cell-density state, inverts each of these. Two design rules follow. First, QS-directed prophage-derived reagents are best matched to a defined pathogen and a defined disease mechanism, for example T3SS1-dependent cytotoxicity or biofilm accumulation on hatchery surfaces, rather than deployed as broad anti-virulence agents. Second, efficacy endpoints must be output-specific, since MIC and MBC values do not capture anti-virulence activity and a compound that lowers one virulence readout may raise another. This constraint also bears on how resistance should be assessed: anti-virulence agents impose weaker selection than bactericides, but selection is not absent, and regulatory mutants that escape QS control are a predictable escape route.

## 7. Applications in Aquaculture

Four settings are considered for applying the mechanistic toolkit described above: treatment of active vibriosis, hatchery biosecurity, feed-based delivery, and water or surface treatment. The available evidence ranges from laboratory assays and controlled challenge experiments to hatchery-relevant experimental systems. Evidence for routine commercial application of purified prophage-derived products is currently lacking.

### 7.1. Therapeutic Use Against Vibriosis in Finfish and Shellfish

Direct challenge trials have accumulated across the commercially most important host–pathogen pairs. In shrimp, the AHPND/*V. parahaemolyticus* axis has been the primary target. Prophylactic and therapeutic phage administration in *Penaeus vannamei* protects juveniles against lethal AHPND challenge and reduces histopathological damage in the hepatopancreas [138]. A recent trial with an optimized AHPND-phage preparation improved survival dose-dependently in *L. vannamei*, elevated shrimp immune enzymes (acid and alkaline phosphatases, lysozyme, superoxide dismutase, catalase, glutathione peroxidase), and drove transcriptomic activation of TLR4/MyD88 and Nrf2/PPAR pathways, indicating that phage treatment triggers protective immunometabolic adaptations beyond simple bacterial killing [139]. Not every trial has succeeded: a single-phage bioassay in AHPND-challenged *P. vannamei* juveniles reduced mortality by only 4% and highlighted the need for cocktails combined with adjuvants such as toxin binders, a realistic caveat for translating monotherapy from the bench to grow-out [140].

In finfish larvae, phages delivered directly to rearing water reproducibly reduce vibriosis mortality. Zebrafish larvae challenged with *V. anguillarum* and treated with phage lysate (~10^8^ PFU mL^−1^) survived at levels indistinguishable from unchallenged controls [141]. The broad-host-range phage KVP40 significantly reduced or delayed mortality of *V. anguillarum*-challenged Atlantic cod and turbot larvae and also protected against background pathogen mortality in unchallenged controls, indicating efficacy in hatchery-relevant larval models [142]. In gilthead seabream larvae, the novel *V. harveyi* phage vB_VhaS_MAG7 improved survival by up to 20% against a virulent host strain [143].

Shellfish larvae show equally clear responses. In Pacific oyster (*Crassostrea gigas*) larval cultures, the addition of a two- or three-phage cocktail targeting *V. coralliilyticus* eliminated pathogen-associated mortality and significantly improved metamorphosis to spat, with efficacy scaling from 1 mL laboratory cultures to 10 L systems using non-sterile filtered seawater [144]. In hatchery-relevant trials, a five-phage cocktail against *V. coralliilyticus* and *V. tubiashii* reduced larval mortality by up to 91% at 6 days post-challenge, and a refined three-phage cocktail (VCP300) reduced *C. gigas* mortalities by >90% [145].

Phage-derived proteins are following the same trajectory, from a much smaller evidence base. Endolysin Vplys60, encoded by the *V. parahaemolyticus* phage qdv001 and belonging to the same lytic qdvp001 lineage described in Section 4.1, was expressed in *Pichiapastoris* and showed strong amidase activity in *Artemia* challenge trials, enhancing survival while reducing *Vibrio* load [98]. In rotifers and fish larvae, the lytic phage CH20 and the endolysin LysVPp1, applied as two independent agents rather than as a phage and its cognate lysin, reduced loads of *V. alginolyticus*, *V. parahaemolyticus*, and *V. splendidus* without disrupting the broader microbiota [99]. Beyond vibrios, this class already has field-relevant precedents: the synthetic endolysin ClyX-2 achieved 95% survival in *Streptococcus iniae*-infected hybrid striped bass, equivalent to carbenicillin at concentrations as low as 15 μg mL^−1^, demonstrating that a peptidoglycan-hydrolase-based therapy can substitute for antibiotics in a commercial finfish species [146]. A recent review synthesizes the same picture across species: phage therapy in aquaculture is efficacious, self-propagating in water, host-specific, and gentler on the microbiome and immune systems than antibiotics or disinfectants, but administration route, dose, and host physiology still vary widely across studies and prevent consensus dosing guidelines [147]. No experimentally validated prophage-derived molecule has yet been tested in any of these settings, which is the single largest gap between the framing of this field and its evidence.

### 7.2. Hatchery Biosecurity and Potential Biofilm-Control Applications

Hatcheries are the highest-value application niche because *Vibrio* biofilms colonize construction materials (tile, plastic, stainless steel), resist common disinfectants (Section 5.1), and serve as the biological entry point for vibriosis in larval systems. The clearest field-relevant demonstration comes from oyster hatcheries, where repeated additions of *V. coralliilyticus*-specific phage cocktails to larval tanks reduced larval mortality by up to 91% at 6 days post-challenge, which identifies phage cocktails as potential candidates for hatchery biosecurity [145]. This evidence comes from whole virulent phages, and no equivalent hatchery-scale dataset exists for any prophage-derived molecule.

Direct evidence for eradication or sustained suppression of established *Vibrio* biofilms in operational hatchery systems remains limited. Most hatchery-related studies measure pathogen abundance, prevention of colonization, or host survival rather than removal of mature surface-associated biofilms. Evidence for disruption of established biofilms comes mainly from controlled laboratory assays using model surfaces. Long-term control of established *Vibrio* biofilms by experimentally validated prophage-derived molecules has not yet been demonstrated under commercial hatchery conditions.

A recurring concern with hatchery-scale phage application is off-target disturbance of the microbiota. Direct 16S rRNA sequencing of Atlantic salmon (*Salmo salar*) rearing water treated with a *Flavobacterium columnare* phage showed that phage treatment prevented mortality, reduced pathogen relative abundance, and produced only minor community-composition shifts in the presence of the host and no detectable α-diversity change in its absence, whereas oxytetracycline in the same system did not reduce the pathogen at all [148]. Comparable microbiota-safety data have been reported for endolysins and lytic phages applied to rotifer and larval-fish systems, where 16S rRNA analyses showed only limited, context-dependent shifts in community structure and no change in α-diversity in *V. alginolyticus*-treated larvae [114]. These datasets are the strongest evidence available that phage-derived molecules can be deployed in recirculating systems without triggering the dysbiosis routinely produced by antibiotic prophylaxis, although both were generated over short exposures in small systems with lytic agents, so they do not yet address repeated dosing, temperate agents, or commercial-scale recirculation. The residual uncertainty is why microbiome disturbance is retained as an open biosafety question in Section 9.4 rather than treated as resolved. Live-feed pre-treatment is the second hatchery use case. As in Section 5.2, phage cocktails applied directly to *Artemia* or rotifer cultures decontaminate the live prey before it enters the larval tank: the φSt2/φGrn1 cocktail reduced presumptive *Vibrio* in *A. salina* by 93% in 4 h [110], and *Pichia* expressing Vplys60 achieved comparable control in *Artemia franciscana* while enhancing survival [98], illustrating that hatchery biofilm control can begin one trophic level upstream of the fish itself.

### 7.3. Feed-Based Delivery Approaches

Delivering prophage-derived molecules through feed is attractive for scale, cost, and farmer compliance, but oral phage payloads face two obstacles: gastric acid inactivation and premature release into rearing water. Encapsulation in polysaccharide matrices addresses both. An alginate/CaCO_3_ microencapsulation method achieves ~100% phage-loading efficiency, protects phages from gastric acid, releases them in simulated intestinal fluid, and (in a farm-scale broiler *Salmonella* trial) outperforms non-encapsulated phage cocktails in both efficacy and duration [149]. Chitosan-coated alginate microparticles produced by spray drying retain their cargo in acidic conditions and release it in alkaline intestinal pH; delivered orally to tilapia in medicated food, they transit through the stomach unaffected and reach the anterior intestine within 2 to 4 h, with lysozyme cargo retaining activity throughout [150]. In shrimp, alginate microparticles produced by ionic gelation protect encapsulated cargo through the digestive tract of *Penaeus*. Probiotic *Bacillus licheniformis* reached the shrimp intestine at 51.3% viability compared with 27.2% for free cells, a template directly transferable to prophage-derived enzymes [151].

Two additional feed delivery formats are advancing. Edible antimicrobial coatings applied to fish feed pellets (whey protein isolate (WPI) matrices loaded with phages by dip- or spray-coating) reduce phage release in salt water by more than one log PFU pellet^−1^, protect activity through simulated gastric conditions, and produce 3 to 5 log reductions in *Vibrio* and *E. coli* in simulated intestinal digestion, demonstrating a directly scalable feed-additive route [152]. Bioencapsulation via live prey is the second: phage-enriched *Artemia nauplii* successfully transferred *Edwardsiella tarda* phage ETP-1 into zebrafish, establishing that the *Artemia* → larva route works for phage transfer as well as for vaccines and probiotics [153]. These formats will scale most readily to prophage-derived enzymes such as endolysins and depolymerases, whose proteinaceous nature otherwise leaves them vulnerable to gut proteases and salt water in unformulated aqueous suspensions.

### 7.4. Water Treatment and Surface Coatings

Phage-based water treatment sits at the intersection of aquaculture and industrial water reuse. A survey of phage applications in water treatment established the biochemical basis for selectively suppressing problem bacteria without the collateral damage caused by broad-spectrum disinfectants (activated-sludge bulking, resistant strains, biofilm-driven corrosion, and cyanobacterial blooms are all candidate targets), with the added advantage of no disinfection-byproduct generation [154]. Specifically, in recirculating aquaculture systems, seasonal monitoring of viral abundance and phage persistence found that *V. parahaemolyticus* phage survived for roughly two weeks in aquaculture water, while *A. salmonicida* phage persisted for about three months, and repeated additions produced no significant shift in the resident bacterial community, laying the empirical basis for treating rearing water as a continuous, phage-permissive matrix rather than a batch-dosing environment [155].

Membrane bioreactors and ultrafiltration systems used in aquaculture water treatment are the second high-yield target. T4-phage-functionalized ultrafiltration membranes cut biofouling flux losses from 71% to 36% over 6 h, and O_2_-plasma-treated surfaces further enhanced phage retention and biofouling resistance [156]. In a modified polyvinylidene-fluoride/sulfonated-graphene-oxide MBR, adding a lytic phage against an antibiotic-resistant *E. coli* increased permeate flux from 60 to 125 L m^−2^ h^−1^, and the combined membrane-modification-plus-phage strategy reduced both pore-blocking and cake-layer fouling [157]. A quorum-quenching/hitchhiking-phage phoresy system deployed on reverse-osmosis membranes disrupted *P. aeruginosa* biofilm QS signaling, decreased EPS synthesis, restored 90% of membrane flux in severely biofouled systems, and extended membrane lifespan, directly relevant to aquaculture RAS water polishing [158].

For surface coatings (hatchery tank walls, pipework, and infrastructure), several immobilization chemistries have been developed. A recent review compiles the main strategies for anchoring whole phage particles on planar or particulate substrates, including physisorption, covalent linkage, and layer-by-layer assembly [159]. Polyelectrolyte-multilayer coatings functionalized with phages via electrostatic capture on cationic outermost layers deliver significant antibacterial activity in contact assays, with the antimicrobial effect coming entirely from the immobilized phage rather than the polymer scaffold, a design directly transferable to hatchery surface materials that require prolonged, non-eluting antimicrobial function [160]. Combining these surface strategies with the polyvalent *Vibrio*-active depolymerase and endolysin classes (Section 4.2 and Section 4.3) has been proposed for antifouling coatings directed at marine biofilms, although no *Vibrio*-specific surface of this type has been reported at pilot scale.

Finally, phage-derived interventions are increasingly deployed alongside probiotics in live-feed systems, and the combination evidence is evaluated in Section 8.4 rather than repeated here [161]. Combining prophage-derived molecules with probiotics, biosurfactants, quorum quenchers, and nanoparticle carriers is now the dominant translational modality, and every feed, water, and surface delivery format described above is a candidate substrate for that broader combination-therapy pipeline.

## 8. Combination Therapies

Section 4, Section 5, Section 6 and Section 7 established that individual phage-derived molecules, principally endolysins and polysaccharide depolymerases, together with whole lytic phages, can each control *Vibrio* in defined settings, and that experimentally validated prophage-derived agents remain confined to the regulatory class described in Section 4.6. Monotherapy repeatedly encounters the same three obstacles across models and species: bacterial regrowth after initial lysis, a narrow host range that leaves resistant subpopulations untouched, and the impermeable Gram-negative outer membrane that blocks exogenously applied endolysins. Combination strategies address each obstacle by pairing these agents with orthogonal partners (membrane permeabilizers, antimicrobial peptides, antibiotics, probiotics, biosurfactants, quorum quenchers, essential oils, and nanoparticles) and are currently the most actively investigated approach in anti-*Vibrio* development. A recent broad review captures the shared logic: phages and their encoded lytic proteins consistently achieve greater sterilization and lower emergence of resistance when co-administered with sub-MIC antibiotics, with β-lactams showing the most consistent synergy owing to their complementary attack on peptidoglycan [162]. Table 3 summarizes combination-therapy studies, their target species, model systems, evidence maturity, and outcomes; entries drawn from work outside the genus *Vibrio* are labeled as comparative examples and should not be read as *Vibrio* results.

### 8.1. Phage-Antibiotic Combinations

Phage-antibiotic combinations against *Vibrio* have now moved from in vitro checkerboard assays to in vivo efficacy studies. Phage Φ170, isolated from shrimp-farm sewage, produces strong lytic activity against multidrug-resistant *V. parahaemolyticus* on its own; when co-administered with doxycycline in zebrafish challenged with the same pathogen, the phage-antibiotic pair produced synergistic reductions in bacterial burden, restored liver and intestine histology, and dampened excessive inflammatory responses, the first direct demonstration that phage-antibiotic synergy translates into an aquaculture-relevant host [121]. The mechanistic basis for this synergy is well established in other Gram-negative systems: sub-MIC antibiotics slow bacterial replication, filament cells, or perturb the outer membrane in ways that expose more phage receptors and delay resistance emergence, while phage-driven lysis lowers the effective bacterial inoculum to a range at which even reduced antibiotic doses become bactericidal [162]. Two design variables emerge from that literature and remain untested in *Vibrio*. Order matters: in *P. aeruginosa* PA14 biofilms, some phage and drug combinations reduce bacterial density below either single treatment, with a marked order effect in which pre-treatment with phage before the drug maximizes killing, and the phage simultaneously suppresses the ascent of antibiotic-resistant subpopulations [163]. Interval matters as well: sequential protocols applying gentamicin or ciprofloxacin 6 h after phage drove biofilm counts below detection in both mono-species and dual-species systems [164]. Neither the order effect nor the optimal interval has been systematically examined in any *Vibrio* system, and because both are scheduling variables rather than new reagents, testing them is among the cheapest available routes to improved efficacy. From an aquaculture perspective, the pragmatic implication is that phage cocktails may allow substantial dose sparing of licensed antimicrobials, a partial resolution to both antibiotic-residue concerns and resistance-driven treatment failure [165].

### 8.2. Endolysin and Lytic-Protein Combinations with Membrane Permeabilizers

The dominant obstacle to exogenous endolysin activity against *Vibrio* is the Gram-negative outer membrane, and the mainstream solution is co-administration with an outer-membrane permeabilizer. For aquaculture, the permeabilizers that matter most are the non-antibiotic ones, and these are treated first here for that reason. The vibriophage endolysin Lysqdvp001, paired with the food-grade cationic antimicrobial ε-poly-lysine, produced strong synergistic killing of *V. parahaemolyticus*, driving reductions of 3.75, 4.16, and 2.50 log_10_ CFU g^−1^ in cod, shrimp, and oyster tissue respectively and removing 44 to 68% of preformed biofilms on polystyrene, glass, and stainless steel, through membrane hyperpolarization and enhanced enzyme leakage rather than peptidoglycan hydrolysis alone [61]. Plant-derived permeabilizers offer a second non-antibiotic route: cinnamaldehyde potentiates the endolysin LysK04 (FICI 0.13 to 0.76) against carbapenemase-producing Gram-negatives, with more than 5 log CFU/mL reductions and extensive membrane damage visualized by microscopy [166]. That study was performed on *Klebsiella pneumoniae*, *Acinetobacter baumannii*, *E. coli*, and *P. aeruginosa* and is a comparative example; whether the same pairing works in seawater against *Vibrio* is untested, and the one study that asked this question directly returned a negative answer. KZ144 and LysPA26 tested with EDTA or oregano essential oil against *V. parahaemolyticus* in natural seawater showed no synergy, and the proteinaceous endolysin was consumed as a growth substrate by the fast-growing pathogen [56]. Combination selection is therefore a matrix-specific and species-specific engineering problem rather than a transferable recipe, and marine matrix testing should be a condition of reporting rather than an afterthought.

The most fully characterized *Vibrio* combination to date uses an antibiotic partner, and its regulatory position needs to be stated alongside its efficacy. LysMD30, the first vibriophage-derived L-alanyl-D-glutamate peptidase, hydrolyses peptidoglycan but lacks intrinsic bactericidal activity; combined with the polymyxin colistin it produces broad-spectrum synergy across *V. parahaemolyticus*, *V. harveyi*, and *V. alginolyticus* (FICI < 0.5), lowers colistin MICs fourfold, reduces biofilm viable counts on polystyrene and stainless steel by more than 3 log within 1 h, and confers 100% protection in a *Galleria mellonella* challenge while delaying the emergence of colistin resistance during serial passage [62]. Mechanistic dissection with a related lysin–colistin pair identified a threshold colistin concentration (0.2 to 0.8 μM, depending on LPS density) that permeabilizes the outer membrane enough for the lysin to reach peptidoglycan; beyond that threshold, lysin-driven peptidoglycan hydrolysis facilitates colistin-mediated cytoplasmic-membrane disruption, elevated intracellular ROS, and biofilm dispersal, closing an amplification loop that neither agent completes alone [167]. Independent lysin–colistin pairings (ElyA1 with colistin against *A. baumannii*, *P. aeruginosa*, and *K. pneumoniae*) recapitulate the same pattern in vitro and in murine infection models [168]. Against this, colistin is a highest-priority critically important antimicrobial for human medicine [63], plasmid-mediated *mcr* resistance is attributed substantially to agricultural use [64], and colistin has been withdrawn as a feed additive in China and restricted in the European Union. Deploying a colistin-dependent enzyme in food-animal production would run against the One Health rationale for replacing antibiotics in aquaculture in the first place. The defensible reading is that the lysin–colistin work establishes the permeabilization mechanism and the dose-sparing principle, and that translation should proceed through the food-grade and plant-derived permeabilizers and the antimicrobial peptides discussed above and in Section 8.3, with the same reasoning applying more mildly to the gentamicin [17] and doxycycline [121] combinations reported elsewhere in this review.

### 8.3. Endolysin-Antimicrobial Peptide Combinations

Antimicrobial peptides (AMPs) are the second natural partner for endolysins, exploiting the same “permeabilize-then-lyse” logic without the resistance-selection footprint of legacy antibiotics. T7 endolysin, combined with polymyxin B and colistin, synergistically eradicated *P. aeruginosa* biofilms, and T4 endolysin, combined with nisin, produced synergy against *S. aureus* biofilms, establishing lysin-AMP pairing as a broadly applicable strategy for biofilm eradication that is directly portable to vibriophage endolysins [169]. For vibriophage-derived endolysins in particular, the mini-review of the Lysqdvp001 family notes AMP and permeabilizer partnerships as the most likely near-term route to overcoming the *Vibrio outer*-membrane barrier at industrial scale [7].

### 8.4. Phage-Probiotic and Phage-Biosurfactant Combinations

Probiotic bacteria are an established aquaculture intervention, and combining them with prophage-derived agents pairs anti-adhesion and colonization-resistance mechanisms with direct lytic activity. In non-axenic *Tetraselmis suecica* microalgae and *Artemia salina* live-feed cultures, the broad-host-range vibriophage KVP40 combined with *Phaeobacter inhibens* DSM17395 reduced *V. anguillarum* one to two orders of magnitude below inoculum and reduced *Artemia mortality* more than either agent alone, although in that specific matrix the incremental gain over probiont-only treatment was marginal, again arguing for careful matrix- and dose-optimization rather than off-the-shelf mixing [161]. Biosurfactants isolated from marine bacteria (MSI-A 07 and MSI-A 08) disrupt biofilms of *V. harveyi*, *V. alginolyticus*, *V. vulnificus*, *V. fischeri*, *V. parahaemolyticus*, and *Photobacterium damselae* under dynamic conditions, and their surface-active mode of action is orthogonal to phage lysis, supporting biosurfactant-incorporated feed as a complementary chassis for phage or endolysin delivery in shrimp aquaculture [104]. Phyllosphere-derived antibiofilm compounds paired with vibriophages produced measurable but species-dependent biofilm inhibition, with much stronger effects on *B. cereus* than on *V. harveyi*, a reminder that combination selection must be tuned to the target pathogen rather than assumed to be additive [170].

### 8.5. Phage-Quorum Quencher, Phage-Essential Oil, and Phage-Nanomaterial Platforms

Quorum-quenching organisms and enzymes constitute a third orthogonal class of partners. A hitchhiking phoresy system, in which the QQ bacterium *Paenarthrobacter nicotinovorans* carries lytic phages onto a *P. aeruginosa* biofilm, simultaneously downregulates QS-driven EPS synthesis and enables phage penetration to the biofilm interior, restoring 90% membrane flux in severely biofouled reverse-osmosis systems and extending membrane lifespan [158]. Applied to *Vibrio*-driven biofouling in RAS water polishing, the same architecture would combine the QS-disruption mechanisms described in Section 6 with the biofilm-degrading depolymerase activity described in Section 4.

Essential oils are a further class of adjuvants. A recent conceptual synthesis reframes EOs not as standalone antimicrobials but as biophysical adjuvants (EO-driven membrane disruption primes bacteria for phage predation by exposing receptors and steering populations toward more susceptible phenotypes), although the field remains largely in vitro with limited preclinical validation [171].

Nanomaterial platforms are the fastest-growing combination modality. Nanoparticles stabilize phage particles, extend circulation and shelf-life, and enable photothermal or photodynamic co-therapies; in return, phages endow nanomaterials with species-specific bacterial recognition, reducing off-target microbiota destruction [172]. Encapsulation formats specifically developed for phage delivery (liposomes, polymeric nanoparticles, hydrogels, and nanofibers) extend phage half-life in systemic circulation and enable site-specific release, and directly complement the alginate and chitosan carriers already validated in aquaculture (Section 7.3) [173]. Silver-chitosan nanoparticles combined with phages produced synergistic killing of Gram-positive and Gram-negative pathogens and reduced MICs several-fold relative to either agent alone, extending to biofilm formation as well [174].

A related development is the isolation of vibriophages that disrupt biofilms outside their productive host range, described in Section 4.2 and Section 5.3: applied as a cocktail, VpT and VpR degrade preformed biofilms of eight non-host *Vibrio* species and complex multi-species communities through distinct infection strategies that together suppress bacterial regrowth [72]. In effect, a well-designed phage cocktail can itself be a combination therapy, with each phage contributing an orthogonal mechanism such as adsorption, presumptive depolymerase activity, or lysis timing, and this internal combinatorial logic is emerging as a pragmatic path to broad-spectrum *Vibrio* biofilm control without importing exogenous antimicrobial partners. The cocktail literature is not uniformly positive, and the exceptions are informative: in one *A. franciscana* bioassay, the individual phages TDD and SRI outperformed their cocktail in log-CFU reduction [175], showing that combining phages is not additive by default and that cocktail composition requires the same matrix-specific optimization demanded of chemical partners.

No single prophage-derived molecule solves the composite *Vibrio* problem of biofilms, resistance, and outer-membrane refractoriness on its own, but carefully paired combinations routinely achieve efficacy that neither partner reaches in isolation. The immediate translational challenges are formulation stability, dosing kinetics, the regulatory pathway, and the failure modes documented in the natural-seawater endolysin work, all of which are taken up in Section 9.

**Table 3:** Combination-therapy studies, including partner agent, target species, model system, evidence maturity, and outcome. Evidence maturity is assigned by the authors and given at the start of the model column using a fixed vocabulary: in vitro only; invertebrate model; fish or shellfish challenge; hatchery scale. Entries performed outside the genus *Vibrio* are labeled comparative example and report no *Vibrio data*.

Combination	Target *Vibrio*	Model or Matrix	Effect	Reference
Phage Φ170 + doxycycline	Multidrug-resistant *V. parahaemolyticus*	Fish challenge. Zebrafish challenge model (immersion administration; prophylactic and therapeutic arms)	Synergistic reduction in bacterial burden, restoration of liver and intestine histology, and suppression of excessive inflammatory responses; Φ170 alone conferred strong lytic activity and prophylactic survival benefit	[121]
Endolysin LysMD30 + colistin	*V. parahaemolyticus*, *V. harveyi*, *V. alginolyticus*	In vitro plus invertebrate model. Checkerboard MIC; biofilms on polystyrene and stainless steel; *Galleria mellonella* infection	Broad-spectrum synergy (FICI < 0.5); 4-fold reduction in colistin MIC; >3 log reduction in biofilm viable counts on polystyrene and stainless steel within 1 h; 100% *Galleria protection*; delays colistin-resistance emergence. Colistin is a WHO highest-priority critically important antimicrobial, which constrains use in food-animal production (Section 8.2)	[62]
Endolysin Lysqdvp001 + ε-poly-lysine (ε-PL)	*V. parahaemolyticus*	In vitro, food matrix. Seafood matrices: *Gadus macrocephalus* (cod), *Penaeus orientalis* (shrimp), oyster tissue; biofilms on polystyrene, glass, stainless steel	3.75, 4.16, and 2.50 log_10_ CFU/g reductions in cod, shrimp, and oyster tissue, respectively; 44 to 68% removal of preformed biofilms on polystyrene, glass, and stainless steel; membrane hyperpolarization and enhanced enzyme leakage	[61]
Vibriophage KVP40 (broad host range) + probiotic *Phaeobacter inhibens* DSM17395	*V. anguillarum*	In vitro plus invertebrate model. Non-axenic *Tetraselmis suecica* microalgae and *Artemia salina* live-feed cultures; CHSE-214 fish cell line	One to two orders of magnitude reduction in *V. anguillarum* below inoculum in *T. suecica* cultures; lower *V. anguillarum* with the combination than probiont alone; immediate killing of *V. anguillarum* in *Artemia*; incremental gain over probiont-only was marginal in *Artemia* matrix	[161]
Endolysins KZ144 and LysPA26 + EDTA or oregano essential oil (outer-membrane permeabilizers) in natural seawater	*V. parahaemolyticus* ATCC-17802	In vitro only. Natural seawater bactericidal assays	Endolysins retained muralytic activity in seawater but did not synergize with EDTA or oregano EO; after permeabilizer action, *V. parahaemolyticus* metabolized the endolysins as a growth substrate, a negative result flagging matrix-specific failure of the permeabilizer + endolysin strategy	[56]
Vibriophage cocktail VpT + VpR (rationally paired distinct-mechanism phages)	Multidrug-resistant *V. parahaemolyticus* (host); 8 non-host *Vibrio* species and complex multispecies communities	In vitro only. Preformed biofilm degradation; cross-host-range biofilm assays; polymicrobial biofilm communities	Cocktail degraded preformed biofilms of 8 non-host *Vibrio* species and complex multispecies *Vibrio* communities via distinct infection strategies; synergistically suppressed bacterial regrowth, internal-combinatorial phage cocktail as a self-contained combination therapy	[72]
Three genome-mined phage endolysins + gentamicin	*V. parahaemolyticus* (63 clinical, food, and environmental isolates)	In vitro only. Preformed biofilms; combination-of-inhibitor testing across a broad isolate panel	Synergistic combination with gentamicin; cleared >90% of preformed biofilms within 48 h across a broad *V. parahaemolyticus* panel	[17]
Endolysin LysK04 + cinnamaldehyde (plant-derived outer-membrane permeabilizer)	Not tested against *Vibrio*; validated on carbapenemase-producing Gram-negatives (*K. pneumoniae*, *A. baumannii*, *E. coli*, *P. aeruginosa*). Comparative example	In vitro only, comparative example. Checkerboard assays; time-kill assays; FESEM	Synergistic/additive interactions (FICI 0.13 to 0.76); >5 log CFU/mL reduction in time-kill assays; biofilm disruption; extensive structural damage in co-treated cells, supports phyto-enzyme combinations as a translational template for *Vibrio* biofilm control	[166]
T7 endolysin + colistin/polymyxin B; T4 endolysin + nisin	Not tested against *Vibrio*; cross-species Gram-negative and Gram-positive biofilms. Comparative example	In vitro only, comparative example. Recombinant endolysin + AMP checkerboard; biofilm eradication assays	T7 endolysin combined with polymyxin B and colistin synergistically eradicated *P. aeruginosa* biofilms; T4 endolysin with nisin synergized against *S. aureus* biofilms; framework generalizes to vibriophage endolysin + AMP pairing	[169]
Marine biosurfactants (lipopeptides from MSI-A 07, MSI-A 08), adjuvant to phage/endolysin therapy	*V. harveyi*, *V. alginolyticus*, *V. vulnificus*, *V. fischeri*, *V. parahaemolyticus*, *Photobacterium damselae*	In vitro only. Dynamic biofilm assays on multiple shrimp pathogens	Both lipopeptide biosurfactants disrupted biofilm formation under dynamic conditions across six *Vibrio* species, supporting biosurfactant-incorporated feed as a complementary chassis for phage or endolysin delivery in shrimp aquaculture	[104]
Phyllosphere-bacterial supernatants (*Pseudomonas fluorescens* JB 3B and *Proteus myxofaciens* JB 20B) + vibriophage Vh-S4	*V. harveyi* (biofilm)	In vitro only. Antibiofilm assays; SEM	Species-dependent effects: *V. harveyi* biofilm inhibition 3.5 to 4.8% and destruction 6.4 to 9.3% only, much weaker than the same combinations against *B. cereus* (~39 to 50%), showing the need for pathogen-specific optimization rather than universal cocktails	[170]
Quorum-quenching bacterium (*Paenarthrobacter nicotinovorans*) carrier + hitchhiking lytic phages (‘phage phoresy’ architecture)	Not tested against *Vibrio*; applied to *P. aeruginosa* reverse-osmosis membrane biofouling. Comparative example	Pilot scale, comparative example. Off-site RO membrane cleaning; long-term operation	QQ downregulated QS-driven EPS synthesis while phages penetrated and lysed biofilm interior; 90% flux recovery in severely biofouled RO membranes; extended membrane lifespan; template for QS-plus-phage biofilm control of *Vibrio* in aquaculture water polishing	[158]
Phage cocktail (Eric + Ariel; nucleus-forming Chimallinviridae vibriophages)	*V. parahaemolyticus* AHPND strains	Shellfish challenge. In vitro regrowth kinetics; in vivo shrimp AHPND challenge	Two nucleus-forming phages, Eric and Ariel, with a wide host spectrum against VP_AHPND; the cocktail prolonged bacterial regrowth of multiple pathogenic VP_AHPND strains and reduced shrimp mortality from VP_AHPND infection	[176]
Five-phage cocktail (novel siphoviruses), internal-combinatorial monotherapy vs. individual phages	Pathogenic *Vibrio* sp. Va-F3 (shrimp vibriosis)	Shellfish challenge. In vitro growth-inhibition assays; in situ shrimp survival trial	Cocktail more efficient at inhibiting Va-F3 than any single phage in vitro; shrimp survival was 91.4% (cocktail) vs. 91.6% (antibiotic) vs. 20.0% (untreated) at 7 days. The phage cocktail matched antibiotic efficacy in vivo	[177]
Three-phage cocktail (*V. coralliilyticus*-specific) vs. mono-phage treatment	Multiple-antibiotic-resistant and phage-resistant *V. coralliilyticus*	Shellfish challenge. Cell-lysis test with MAR/PR variants; in vivo Pacific oyster (*C. gigas*) larvae	Cocktail produced an excellent bactericidal effect against MAR and phage-resistant variants, in contrast to two mono-phage treatments (pVco-5, pVco-7); in vivo trial confirmed preventive effect in Pacific oyster larvae against MAR/PR *V. coralliilyticus*	[178]
Two-phage cocktail vB_Vc_SrVc2 + vB_Vc_SrVc9 (broad-host-range vibriophages)	Multiple pathogenic *Vibrio* species	Shellfish challenge. Pacific white shrimp (*L. vannamei*) postlarvae; culture-dependent + 16S microbiome analysis; histopathology of hepatopancreas	Selective and significant reduction in *Vibrio* species after cocktail administration; less hepatopancreas damage and higher lipid accumulation in B cells; provides an environmentally safe alternative to antibiotic prophylaxis	[179]
Two-phage cocktail T2A2 + VH5e	AHPND-causing *V. parahaemolyticus* (VPAHPND)	Invertebrate model. Lytic assays across AHPND-outbreak strains; brine shrimp experimental infection	Phage mixture significantly increased brine shrimp survival vs. untreated groups (*p* < 0.001) against three highly virulent VPAHPND strains; harmless to test organisms, supports phage cocktails as a candidate AHPND control	[180]
Two-phage cocktail phage TDD (vB_VaC_TDDLMA) + phage SRI (vB_VaC_SRILMA)	*V. alginolyticus* (host strain *V. alginolyticus* CECT 521)	Invertebrate model. Artificial marine water + *A. franciscana* bioassay; microbiota impact assessment	Single phages outperformed the cocktail in log-CFU reduction (TDD 4.3, SRI 2.1, cocktail 1.9 log CFU/mL after 12 h), but the cocktail matched SRI in *Artemia* reduction (85.3% vs. 87.3%); no impact on *A. franciscana* natural microbiota or non-target *Vibrio*, a case where the cocktail did NOT improve on the best single phage	[175]
Three-phage cocktail (Myoviridae VHM1, VHM2 + Siphoviridae VHS1)	*V. harveyi* (also lyses *V. alginolyticus* and *V. parahaemolyticus* of the *harveyi* clade)	Invertebrate model. *Penaeus monodon* larval microcosm (*V. harveyi* challenge at 10^5^ CFU/mL; phages at 10^9^ PFU/mL)	Enhanced *P. monodon* larval survival at 96 h vs. untreated control; broad *harveyi*-clade coverage supports eco-friendly *Vibrio*-disease control in shrimp aquaculture	[181]
Silver-chitosan nanoparticles (Ag-CS-NPs) + phage	Not tested against *Vibrio*; Gram-positive and Gram-negative pathogens including biofilm-forming isolates. Comparative example	In vitro only, comparative example. MIC/MBC assays; biofilm-formation inhibition	Combination reduced MIC 2- to 4-fold vs. single agents; enhanced bactericidal killing at 16.5 to 30.1 μg/mL Ag-CS-NPs with phages; effective against Gram-negative biofilms and antibiotic-resistant strains	[174]

## 9. Challenges and Future Perspectives

The evidence cataloged in Section 4, Section 5, Section 6, Section 7 and Section 8 supports a narrower claim than is often made for this field. Phage-derived endolysins and one validated polysaccharide depolymerase are effective anti-*Vibrio* agents in defined laboratory and hatchery settings, mostly in combination and mostly from virulent phages, while experimentally validated prophage-derived molecules remain confined to regulatory modules that have not been formulated or dosed. Translating any of these into routine aquaculture practice requires resolving five interlocking challenges: protein and formulation stability, evolution of resistance and host defense, laboratory-to-farm scale-up, regulatory and biosafety oversight, and the engineering pathway to next-generation agents.

### 9.1. Stability, Formulation, and Delivery of Prophage-Derived Proteins

Recombinant endolysins are proteins, and most native endolysins have short shelf-life expectancies and poor tolerance to temperature, salt, and pH extremes, a well-known limitation for translational application. The streptococcal endolysin PlyC illustrates the general problem: despite its outstanding bacteriolytic activity, it is relatively unstable and requires bioengineering to achieve industrial-scale shelf life. Rational design and directed evolution mutations (T406R and N211H) additively increased thermodynamic stability by 7.5 °C and kinetic stability by roughly 30-fold, establishing that endolysin thermostability can be engineered rather than inherited [182].

Naturally polyextremotolerant endolysins provide the second route. LysBT1, encoded by a prophage of the thermophile *Brevibacillus thermoruber* WF146, retains >60% activity after 1 h at 95 °C and is active from pH 4 to 11, properties driven by a C-terminal S-layer homology (SLH) domain that mediates trimerization, cell-surface binding, and stabilization at elevated temperature [183]. The thermostable prophage endolysin TP84_28 from a *Geobacillus stearothermophilus* phage retains lytic activity at 77.6 °C and trace activity after 30 min at 100 °C, supporting its use as a mild surface disinfectant for high-temperature aquaculture and food-processing infrastructure [184]. Marine-derived vibriophage endolysins tolerate the salinity and temperature regimes relevant to aquaculture. LysMD30, for example, retains 37% activity at 85 °C, >90% activity at pH 5.0 to 8.0, and >60% activity at 800 mM NaCl [62], a favorable starting point that reduces the engineering burden compared with endolysins for clinical applications.

Formulation is the second half of the stability problem. As cataloged in Section 7.3, alginate/CaCO_3_ and chitosan-alginate microparticles protect cargo from gastric acid and release it at intestinal pH, and edible whey-protein-isolate coatings extend feed-pellet phage viability by more than one log during storage, templates directly transferable to endolysin cargo. What still needs work in the *Vibrio*, specifically, is quantitative shelf-life data at temperatures and salinities that match tropical hatchery conditions, and standardized comparisons among free protein, encapsulated protein, and whole-phage formulations under the same storage regime.

### 9.2. Resistance Development and Evolutionary Considerations

Bacterial resistance to lytic phages is inevitable under sustained selection, but for prophage-derived interventions the evolutionary picture is more nuanced than for chemical antimicrobials, and often favorable. Evolved *E. coli* resistant to lytic phages pay context-dependent fitness costs: resistance is advantageous under strong phage pressure but imposes a measurable fitness cost in the phage’s absence, and the magnitude of that cost varies with environmental conditions such as temperature [185]. In *K. pneumoniae*, receptor-modification resistance to capsule-targeting phages either mutates the capsule biosynthesis cluster or alters exopolysaccharides, converting cells to mucoid variants that are cross-resistant to the same-receptor phages but sensitized to phages using alternative receptors, and the selection pressure that produces this resistance also drives expulsion of a multidrug-resistance plasmid, effectively re-sensitizing the population to antibiotics [186]. The same pattern recurs across pathogens: *Salmonella* Typhimurium exposed to phage PBST10 becomes more susceptible to ampicillin with suppressed *tolC* efflux [187]; *Salmonella* Enteritidis mutants resistant to a rationally designed four-phage cocktail using distinct LPS and outer-membrane receptors show significantly reduced virulence and enhanced antibiotic susceptibility relative to single-phage-resistant mutants, translating into stronger in vivo therapeutic efficacy [188]. In *Ralstonia solanacearum*, phage selection produced small-colony variants with mutations in the QS receptor *phcS* and pilus-encoding *pilD* that combined phage resistance with loss of biofilm formation, twitching motility, and virulence *in planta*, the same virulence apparatus identified as a therapeutic target in Section 6 [189].

For *Vibrio* aquaculture, the practical implications are twofold. First, phage cocktails should be rationally assembled from phages targeting different surface receptors, so that the resistance trajectory forces bacteria to sacrifice multiple structural or metabolic assets simultaneously, the same “internal-combinatorial” logic identified in Section 8.5 for VpT/VpR. Second, phage-driven receptor loss can be genuinely deleterious to virulence, so incomplete pathogen eradication is not necessarily therapeutic failure: the surviving population is often less pathogenic and more sensitive to co-administered antibiotics.

A factor absent from most of the aquaculture phage literature, including the studies summarized above, is the bacterial anti-phage defense repertoire. Vibrios carry CRISPR-Cas systems, and evidence of active host-phage conflict appears twice in the prophage catalog compiled in Section 2.2, where a CRISPR spacer in *V. anguillarum* PF7 matches the zot gene of an inovirus resident in strain PF4 [19], and two CRISPR-spacer matches were recovered for the *V. campbellii* prophage HY01 [43]. Beyond CRISPR, seventh-pandemic *V. cholerae* carries the *DdmABC* and *DdmDE* modules on two pathogenicity islands, and *DdmABC* defends against phage infection by triggering abortive infection [190]. Three consequences follow. Whole-phage and engineered-phage approaches will encounter strain-level defense variation that is not predictable from host range assays alone, so defense-system genotyping belongs in the per-strain characterization argued for above. Purified enzymes such as endolysins, depolymerases, and tailocins are largely exempt, since they neither inject DNA nor replicate, which is an underappreciated argument in their favor relative to whole-phage therapy. And abortive-infection systems complicate the reading of phage efficacy data, because population-level suppression can occur without productive infection. Systematic surveys of anti-phage defense across aquaculture-relevant *Vibrio* isolates have not been reported and would materially improve candidate selection.

### 9.3. Translation from Laboratory to Commercial Aquaculture

The gap between laboratory efficacy and commercial deployment is documented in a recent critical review of aquaculture phage therapy: efficacy is well demonstrated across host species, but the diversity of administration routes, doses, target strains, and host physiologies has prevented consensus dosing guidelines, and clear cost-effectiveness comparisons against vaccines and traditional antimicrobials are still lacking [147]. A second review focused specifically on translation identifies the same barriers (receptor-mutation resistance, quorum-sensing modulation of susceptibility, environmental stressors, absence of standardized protocols, unresolved safety-assessment frameworks, and unclear economic feasibility) and further notes that genetically modified phages, while offering more precision, raise additional ethical and ecological concerns [191]. Beyond the biological questions, veterinary-medicine-focused analysis highlights the manufacturing side: manufacturing phage-based products under existing chemotherapeutic rules is unlikely to be commercially viable, and product classification (veterinary medicinal product, magistral preparation, food additive, or biocide) substantially changes the regulatory and market pathway [192]. The economic question deserves to be posed more precisely than it usually is. Recombinant enzymes are priced per unit mass of purified protein, whereas hatchery interventions are costed per unit volume of rearing water or per production cycle, and no published study reports an enzyme dose per cubic meter alongside a cost of goods, so the comparison against chlorine-based disinfection or prophylactic antibiotic use cannot currently be made at all. Until candidate enzymes are reported with an effective concentration in a defined water volume, a required dosing frequency, and a production titer, economic feasibility will remain an assertion. The immediate translational priorities that emerge are therefore (i) standardized, hatchery-relevant efficacy trials with defined dosing, (ii) validated repositories of well-characterized *Vibrio*-active phages and phage-derived proteins with documented parent-phage lifestyle, (iii) production processes that scale, and (iv) reporting conventions that make cost per treated volume computable.

### 9.4. Regulatory Considerations and Biosafety

Phage medicinal products are difficult to regulate because their biological properties (self-propagation, host-dependent replication, cocktail composition, and evolution during use) do not align with the pharmaceutical-quality, preclinical, and clinical guidance intended for small molecules or biologics. The European Pharmacopeia implemented harmonized quality criteria for phage therapy medicinal products and active substances for the first time in 2024, and new German medical-research legislation aims to open specific regulatory pathways; the anticipated breakthrough (RCT-demonstrated efficacy) has not yet been achieved, although the regulatory framework needed to assess such evidence is now more developed [193]. In the United States, roughly 90 phage clinical trials are ongoing globally, with 41 in the US, and the current FDA framework provides workable (if not yet standardized) paths through investigator-initiated INDs and emergency-use authorizations for compassionate cases [194].

The veterinary and aquaculture pathway is less mature. The IABS phage-veterinary workshop noted that EMA and EDQM guidelines now offer some flexibility to accommodate the biological nature of phages, but that manufacturing feasibility under conventional veterinary-chemotherapeutant rules remains the primary financial barrier, and that regulatory harmonization and pathway convergence are prerequisites for market access [192]. Biosafety concerns specific to prophage-derived molecules include the risk of horizontal transfer of virulence or antimicrobial-resistance genes when temperate phages are used therapeutically, the potential for phage-neutralizing antibody production in vertebrate hosts, and dysbiosis of aquaculture microbiomes, the last of which the direct 16S-rRNA data reviewed in Section 7.2 argue is minimal for both lytic phages and endolysins under hatchery conditions [147].

#### Biosafety and Genomic Screening of Prophage-Derived Candidates

The dual-use problem identified at the end of Section 3 requires an explicit answer, because the prophage compartment that supplies candidate molecules is also the principal route by which toxin genes, resistance determinants, and biofilm-promoting cargo move between vibrios [19,30,35,39,45]. Three risks are specific to this class. The first is carryover of mobile elements or virulence-associated sequences into a recombinant preparation. The second is induction of resident prophages in treated systems, which is not hypothetical: sub-inhibitory concentrations of fluoroquinolones, aminoglycosides, tetracyclines, and chloramphenicol induce the SOS response in *V. cholerae* [26], and the SOS regulon in the *Vibrionaceae* is linked to mobilization of resistance determinants [27], so any protocol that pairs a prophage-derived agent with a sub-MIC antibiotic (Section 8.1 and Section 8.2) is also a prophage-induction protocol. The third is environmental release, which matters most in flow-through and partial-reuse farms that discharge to coastal water, where induced particles and their cargo would enter a natural *Vibrio* community already documented as a site of active phage-mediated exchange [19,20,45].

A screening cascade addresses the first risk at the design stage. The sequence set out below is offered as a practical set of checks that can be applied with existing tools, not as a validated standard, since no harmonized biosafety criteria have yet been established for recombinant antimicrobial proteins intended for aquaculture use. First, predict and delimit the source element in the host genome using automated prophage predictors such as PhiSpy [195], together with at least one independent tool, since boundary calls differ substantially between methods, and record any disagreements rather than reporting a single consensus. Second, explicitly assign lifestyle by searching the delimited element for integrase, excisionase, recombinase, and repressor or antirepressor modules, as well as attachment sites, and report the evidence rather than the label alone, so that downstream users can reassess it. Third, screen the element against curated virulence-factor and antimicrobial-resistance catalogs, excluding any candidate whose source element carries ctxAB, zot, ace, ADP-ribosylating toxin homologs, or mobile resistance determinants. Fourth, express only the single candidate open reading frame in a heterologous host, and characterize the resulting preparation with respect to absence of the source phage genome, absence of infectious particles, residual host nucleic acid, and endotoxin content, the last being relevant because expression in and lysis of Gram-negative hosts releases lipopolysaccharide. No consensus acceptance values exist for any of these parameters in this product class, and limits drawn from human biologics or from industrial enzyme preparations should not be transferred uncritically to an agent applied to a cultured aquatic species; acceptance criteria will therefore need to be set case by case, against the target species, the route of administration, and the exposure regime. Fifth, monitor treated systems for induction of resident prophages, particularly where antibiotic partners are co-administered.

This cascade also supports the use of purified proteins rather than whole phages. A purified recombinant protein carries no integrase, no packaging machinery, and no capacity for lysogenic conversion, so it separates the useful cargo of the prophage compartment from the genetic mobility that makes prophages hazardous. Whole temperate phages do not offer that separation and are not, on the evidence available, an appropriate therapeutic format for aquaculture systems. Two residual questions remain open and should not be presented as settled: repeated protein dosing in fish and shellfish raises immunogenicity and residue questions that have not been studied for any vibriophage endolysin, and the microbiome data reviewed in Section 7.2 cover short exposures with lytic agents in small systems rather than repeated dosing at commercial scale. Genetic containment is only one dimension of biosafety, and a complete assessment for this class will also need to address the potential toxicity of the purified protein to the cultured species and to non-target organisms in the same system, immunogenicity and residue behaviour under repeated dosing, the persistence and degradation of an applied protein in seawater and sediment, compatibility with the host and with its resident microbiota, and the wider ecological consequences of routine application in open or partially open production, alongside the regulatory questions set out in Section 9.4. None of these can be resolved from the literature reviewed here, and each requires application-specific experimental assessment in the production system for which a given agent is intended.

### 9.5. Future Opportunities: Engineered Lysins, Chimeric Enzymes, and Synthetic Biology

The most actively pursued engineering approach is expanding endolysin activity against Gram-negative pathogens by genetically breaching the outer membrane rather than pharmacologically. Three distinct engineering strategies are now well validated, all of them comparative examples developed outside the genus *Vibrio*. Artilysins, which are polycationic or amphipathic peptide fusions to a modular endolysin, traverse the outer membrane and achieve a 4 to 5 log reduction in *P. aeruginosa* and *A. baumannii* within 30 min [196]. Innolysins, which are endolysin and phage receptor-binding protein fusions, use the binding specificity of the receptor-binding protein to deliver the endolysin to its peptidoglycan substrate, as exemplified by T5 endolysin/Pb5 chimeras that kill *E. coli* via the FhuA receptor [197]. Lysocins, which are lysin and bacteriocin fusions that hijack Tol- or TonB-dependent translocons, enable periplasmic delivery, and the prototype PyS2-GN4 log-kills *P. aeruginosa* with minimal endotoxin release, disrupts biofilms, outperforms standard-of-care antibiotics, and protects mice from bacteremia [198]. Simpler antimicrobial peptide and endolysin fusions such as cecropin-A-AbEndolysin increase bactericidal activity 2- to 8-fold against multidrug-resistant *A. baumannii* and synergize with β-lactam antibiotics [199], and fusions of the botulinum neurotoxin translocation domain with T5 or LysPA26 endolysins produce broad-spectrum activity across six Gram-negative species [200]. A recent review consolidates these approaches, covering naturally outer-membrane-active endolysins, chemical adjuvants, and protein-engineering fusions, and identifies engineered lysins as the most developed [55]. For *Vibrio* specifically, LysMD30 already exhibits colistin-dependent activity across three species (Section 8.2), and generalizing the artilysin, innolysin, and lysocin architectures to the marine vibriophage endolysin family is a near-term, well-scoped engineering program. It would also remove the colistin dependence that currently constrains the most advanced *Vibrio* combination, which is the strongest practical argument for pursuing it.

Synthetic-biology approaches to whole phages are the second frontier. CRISPR-Cas gene editing, cell-free genome assembly, and homologous recombineering now enable custom RBP swaps that redirect host specificity, insert antimicrobial payloads, and enable precision genome minimization [201]. Synthetic phages engineered to overexpress SOS-network suppressors act as strong adjuvants that increase bacterial killing by quinolone antibiotics by several orders of magnitude in vitro and improve mouse survival in vivo, a general paradigm for turning phages into resistance-suppressing adjuvants [202]. Biological containment strategies: virion-gene-deficient phages that retain phenotypic function but cannot produce progeny provide a route to environmental deployment with reduced ecological footprint [203]. Modern synthetic-phage design integrates CRISPR-Cas editing, cell-free assembly, and increasingly AI-driven design of RBPs, payloads, and genome architecture, and the resulting toolkit is already being applied to redirect specificity and deliver antimicrobial payloads to targeted subpopulations within complex microbiomes [204].

The convergence of engineered endolysins, rationally designed cocktails, synthetic biology, and aquaculture-appropriate delivery formats sets a plausible trajectory for the next 5 to 10 years: broad-spectrum, marine-stable, resistance-steering anti-*Vibrio* biologics that combine one or more of the mechanisms, delivery modes, and combination partners described above. The regulatory infrastructure to accept such products is beginning to materialize; the immediate scientific bottleneck is the lack of RCT-grade efficacy data, which should be the field’s near-term collective priority.

## 10. Conclusions

Prophage- and phage-derived molecules act against pathogenic *Vibrio* species in aquaculture through several distinct mechanisms, but the supporting evidence is narrower than the volume of published work suggests, and the distinction between the two origins matters. Endolysins and one validated polysaccharide depolymerase account for essentially all measured activity against *Vibrio*, and almost all of it derives from virulent phages rather than from prophages. Experimentally validated prophage-derived molecules are currently limited to regulatory elements: the VP882 VqmA/Qtip antirepressor pair and the associated sRNA VpdS, and the RS1 satellite-phage antirepressor RstC, which drives curing of the CTXΦ prophage. Phage-encoded nucleases, holins and spanins, and tailocins have no *Vibrio*-specific molecules at all. Tailocins illustrate this gap most directly, being the one class that is prophage-derived by definition and the one class with no *Vibrio* data. Claims should therefore be restricted to endolysins and depolymerases, several of which are natively active at seawater salinity and hatchery-relevant temperatures, while the remaining classes are stated as research priorities. Where these agents do work, their advantage over antibiotics lies less in bactericidal potency than in their ability to dismantle biofilms and suppress virulence, the two failure modes that antibiotics address poorly. Because phage resistance often incurs fitness costs, incomplete clearance is not necessarily a therapeutic failure: survivors are often less virulent, less biofilm-competent, and more susceptible to co-administered antimicrobials.

The remaining limitations are biological, technical, and regulatory. Three biological constraints are unresolved. Resistance and anti-phage defense systems vary by strain and are not predictable from host-range data. Quorum sensing does not uniformly attenuate virulence, so anti-virulence targeting must be specified per species and per output. Prophage carriage itself modulates biofilm formation in a strain-dependent direction. On the technical side, the outer-membrane barrier is soluble in principle through artilysins, innolysins, lysocins, and permeabilizer pairings, although the one study that tested permeabilizer pairing in natural seawater returned a negative result; delivery formats spanning microencapsulation, feed coatings, bioencapsulated live prey, and surface functionalization have each been demonstrated at hatchery-relevant scale; and efficacy has been shown in commercially important host–pathogen combinations, though almost entirely with whole lytic phages. What is still missing is standardization: defined potency assays, stability data under farm conditions, cost of goods per volume of treated water, biosafety screening criteria applied consistently to candidate source elements, and a veterinary regulatory pathway that accommodates biological agents with variable composition. What the field now requires is coordinated experimental work rather than further proof of concept, together with reporting conventions that make provenance auditable. The priorities are systematic mining of the *Vibrio* prophage catalog with the parent element and its lifestyle documented at source, expression and screening of the predicted depolymerase and nuclease repertoire, the first characterization of a *Vibrio* tailocin and of a prophage-encoded *Vibrio* holin, rational engineering of chimeric and stabilized enzymes that remove the current dependence on colistin, per-strain characterization of phage-host interactions including anti-phage defense systems, and field trials reporting standardized endpoints and dose per unit volume. Prophage-derived agents are unlikely to replace antibiotics outright; their value lies in occupying the biosecurity and biofilm-management roles that antibiotics fill poorly, thereby relieving some of the selective pressure driving resistance in aquaculture, provided that the agents themselves do not depend on critically important antimicrobials to function.

## Data Availability

No new data were created or analyzed in this study. Data sharing is not applicable to this article.

## Abbreviations

AHL	Acyl-homoserine lactone
AHPND	Acute hepatopancreatic necrosis disease
AI-2	Autoinducer-2
AMP	Antimicrobial peptide
c-di-GMP	Cyclic diguanosine monophosphate
CFU	Colony-forming unit
CPS	Capsular polysaccharide
DPO	3,5-Dimethylpyrazin-2-ol
eDNA	Extracellular DNA
EPS	Extracellular polymeric substances
FICI	Fractional inhibitory concentration index
HGT	Horizontal gene transfer
LPS	Lipopolysaccharide
MOI	Multiplicity of infection
OM	Outer membrane
PFU	Plaque-forming unit
PG	Peptidoglycan
PTLB	Phage tail-like bacteriocin
QS	Quorum sensing
RAS	Recirculating aquaculture system
RBP	Receptor-binding protein
RCT	Randomized controlled trial
SOS	SOS DNA-damage response
sRNA	Small regulatory RNA
T3SS	Type III secretion system
T6SS	Type VI secretion system
TCP	Toxin-coregulated pilus
TSP	Tail-spike protein
Vps	*Vibrio* polysaccharide
Zot	Zonula occludens toxin
